# Lignocellulose-Mediated Gel Polymer Electrolytes Toward Next-Generation Energy Storage

**DOI:** 10.1007/s40820-025-01927-6

**Published:** 2025-11-25

**Authors:** Hongbin Yang, Liyu Zhu, Wei Li, Yinjiao Tang, Xiaomin Li, Ting Xu, Kun Liu, Chuanling Si

**Affiliations:** State Key Laboratory of Bio-Based Fiber Materials, Tianjin Key Laboratory of Pulp and Paper, Tianjin University of Science and Technology, Tianjin, 300457 People’s Republic of China

**Keywords:** Lignocellulosic materials, Gel electrolytes, Energy storage devices, Batteries

## Abstract

The pursuit of high energy density and sustainable energy storage devices has been the target of many researchers. However, safety issues such as the susceptibility of conventional liquid electrolytes to leakage and flammability, as well as performance degradation due to uncontrollable dendrite growth in liquid electrolytes, have been limiting the further development of energy storage devices. In this regard, gel polymer electrolytes (GPEs) based on lignocellulosic (cellulose, hemicellulose, lignin) have attracted great interest due to their high thermal stability, excellent electrolyte wettability, and natural abundance. Therefore, in this critical review, a comprehensive overview of the current challenges faced by GPEs is presented, followed by a detailed description of the opportunities and advantages of lignocellulosic materials for the fabrication of GPEs for energy storage devices. Notably, the key properties and corresponding construction strategies of GPEs for energy storage are analyzed and discussed from the perspective of lignocellulose for the first time. Moreover, the future challenges and prospects of lignocellulose-mediated GPEs in energy storage applications are also critically reviewed and discussed. We sincerely hope this review will stimulate further research on lignocellulose-mediated GPEs in energy storage and provide meaningful directions for the strategy of designing advanced GPEs.

## Highlights

- The latest strategies for the construction of lignocellulose-mediated gel polymer electrolytes are summarized.
- The great potential of macroscopic preparation processes and microstructural design of lignocellulose-mediated gel polymer electrolytes are summarized.
- The excellent suitability of the physicochemical structure of lignocellulosic gel electrolytes and energy storage applications is summarized.

## Graphical abstract

**Figure:**
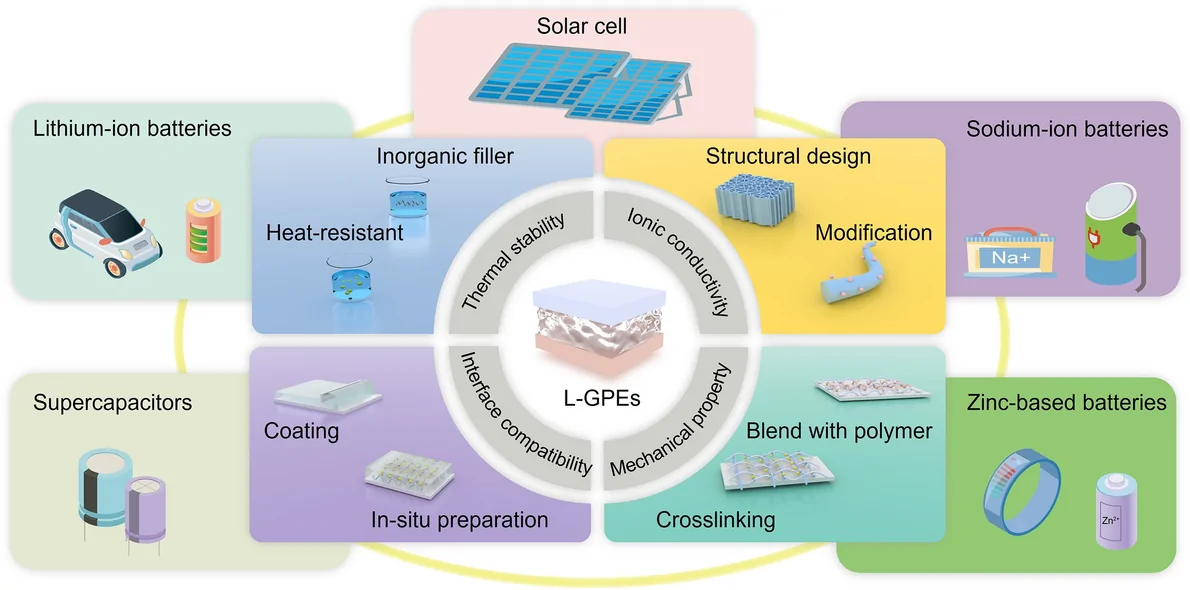

## Introduction

With the rapidly developing modern society, the demand for renewable energy because of the rapid consumption of non-renewable fossil fuels has become a serious issue [1, 2]. The energy demand has driven an unremitting interest in the sustainable development of advanced and environmentally friendly energy storage devices (ESDs) [3]. Electrochemical energy storage device techniques, including supercapacitors (SCs) [4, 5], lithium-ion batteries (LIBs) [6, 7], zinc-ion batteries (ZIBs) [8, 9], sodium-ion batteries (SIBs) [10, 11], and solar cells [12, 13], have received a wide range of attention over the past few decades due to the decreasing demand for energy storage in portable electrical devices, electric vehicles, and grid energy storage systems in electrical equipment and electric vehicles. However, liquid electrolytes, which are widely used in conventional energy storage devices, have become a bottleneck restricting their further development. Liquid electrolytes have some unavoidable safety issues, such as the easy leakage and flammability of organic solvents, as well as some side reactions and dendrite growth [14]. In such situations, solid polymer electrolytes (SPEs) offer hopeful opportunities for effectively addressing safety concerns and stopping dendrite growth in the absence of liquid solvents [15]. Notably, the poor interfacial compatibility, low ionic conductivity (10^–8^-10^–5^ S cm^−1^), and low mechanical properties of solid polymer electrolytes are still challenges for their further deployment [16].

By contrast, gel polymer electrolytes (GPEs) have attracted much attention because they combine the high ion mobility of liquid electrolytes with the safety of solid electrolytes [17]. Generally, GPEs are composed of polymer hosts, liquid electrolytes, and fillers [18]. The polymer host is the backbone of the gel electrolyte, providing the electrolyte with high mechanical support, while the liquid electrolyte dissolves the salts and transports ions, providing high ionic conductivity and superior interfacial stability [19]. Notably, the liquid composition of the swollen polymer body eliminates the problem of organic solvent leakage in electrostatic discharges. Moreover, because of the superior flexibility of the polymer structure, it is possible to physically stop the negative effects of short circuits caused by dendrite growth. As a result, GPEs have emerged as one of the most ideal alternatives in the electrolytes of various electrochemical energy storage devices and have made important progress in the fields of LIBs [20, 21], SCs [22, 23], SIBs [24], LSBs [25, 26], FCs [27], and ZIBs [28, 29]. It is worth noting that traditional synthetic polymer-based GPEs (*e.g*., poly(ethylene oxide), poly(vinylidene fluoride)) still face problems such as insufficient mechanical strength, limited thermal stability, and dependence on petrochemical feedstocks, which make it difficult to meet the dual demands of sustainability and high performance of next-generation energy storage devices.

**Fig. 1:**
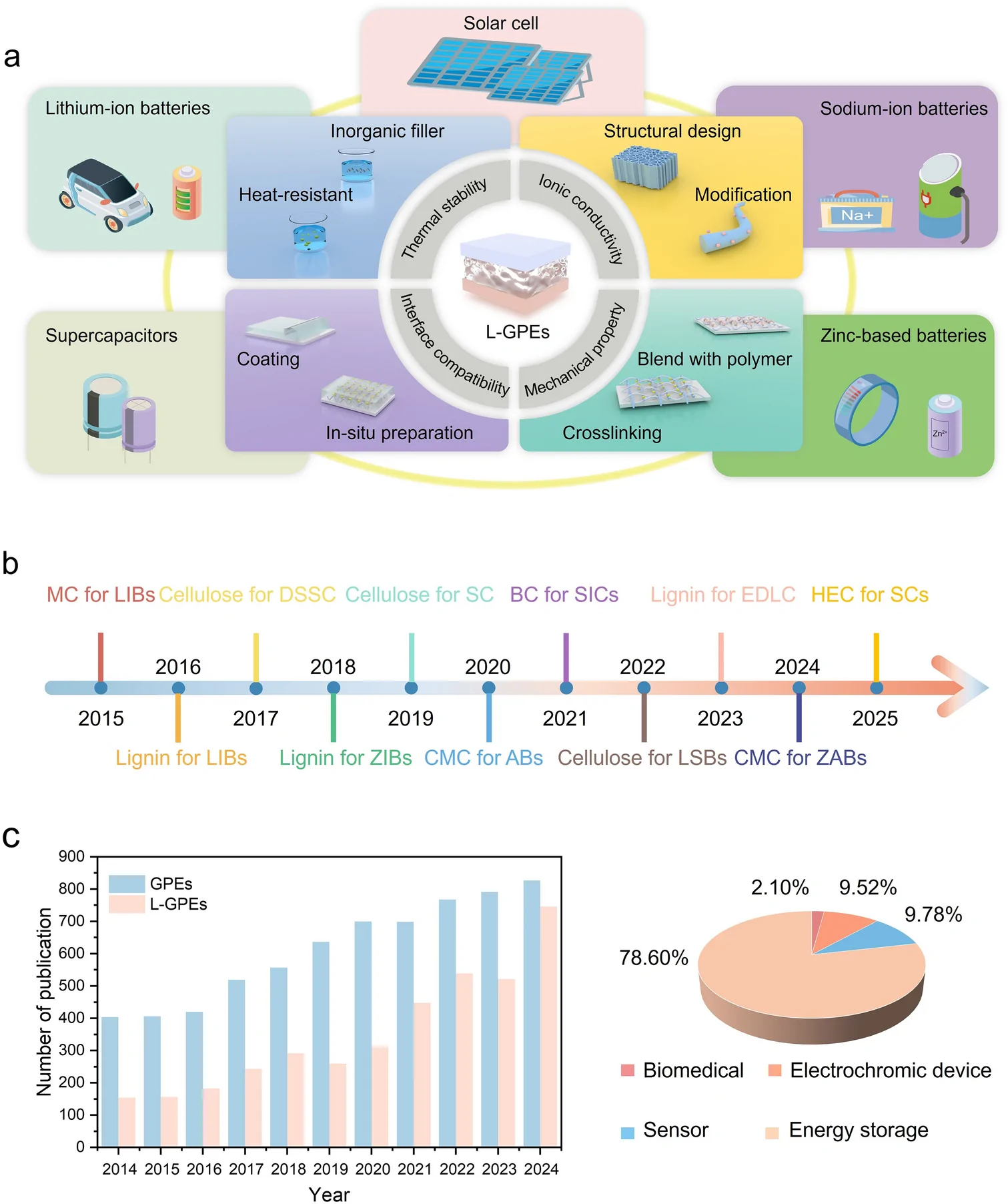
Overview of L-GPEs in energy storage devices. **a** Schematic of the key requirements, preparation method, and energy storage devices applications of L-GPEs. **b** Timeline of the development of representative L-GPEs. **c** Number of publications per year since 2014 regarding the use of L-GPEs according to the Web of Science. And the keywords of publication retrieval are “Electrolytes,” “Cellulose,” “Hemicellulose,” “Lignin”, and “Lignocellulose”. And distribution of publications during 2015–2025 regarding the different GPEs

Therefore, this has driven the exploration of lignocellulosic biomass as an alternative and superior platform for GPE design. Lignocellulose offers a number of unique advantages that directly address the shortcomings of synthetic polymers [30, 31]. As one of the richest renewable resources on earth, lignocellulose has a three-dimensional porous structure, abundant hydroxyl functional groups, and tunable chemical properties, which provide unique advantages for the construction of highly efficient ion transport networks and enhanced mechanical properties [32, 33]. Lignocellulosic materials are mainly composed of cellulose, hemicelluloses, and lignin [34, 35]. In the lignocellulosic material network, cellulose consists of glucose monomers linked by repetitive *β*-1,4 glycosidic bonds with a unique three-dimensional crosslinked porous structure, high crystallinity (60%-80%), and rigid microfibrillar structure (Young's modulus ≈138 GPa) [36]. In gel electrolytes, cellulose acts as a backbone material to provide solid mechanical support for the whole electrolyte system, which makes the compressive strength of L-GPE (> 40 MPa) up to more than 8 times that of PEO-GPE, and physically blocks the path of dendrite growth [37]. Hemicellulose as a branched copolymer composed of different pentoses and hexoses is wrapped around the cellulose, playing a filling and compressive role [38]. The rich branched structure and a large number of hydrophilic groups, such as carboxyl and hydroxyl groups, give hemicellulose an extremely high liquid-absorbing capacity. In gel electrolytes, hemicellulose can absorb a large amount of liquid electrolyte, increasing the ion concentration in the electrolyte, and providing more carriers for ion conduction. At the same time, the absorbed liquid electrolyte fills in the network structure of hemicellulose, forming a continuous ion transport channel, which is conducive to improving ion mobility and conductivity. In contrast, conventional linear polymers (P(VDF-HFP)) need to rely on chain segment creep to transport ions and are prone to crystallization at high temperatures, leading to a sudden drop in conductivity. Lignin is a polyether that consists of different phenolic monomers in different compositions [39, 40]. It cross-links the fiber structure and endows L-GPE with superior thermal stability, with an initial decomposition temperature (> 320 °C) that is 120 °C higher than that of conventional PEO-based GPEs, and a free radical trapping ability that can moreover retard electrolyte combustion [41, 42]. In addition, the hydrophobic three-dimensional crosslinked network of lignin (decomposition temperature > 400 °C) significantly enhances thermal stability and inhibits solvent leakage. This natural multistage structure enables L-GPE to have high mechanical strength and thermal stability at the same time, effectively circumventing the performance shortcomings of traditional GPEs. This combination of sustainability and high performance makes L-GPEs irreplaceable in advanced energy storage devices and a key direction to break through the limitations of traditional electrolyte technology. Due to the unique advantages of lignocellulosic materials, the large-scale production and potential applications of L-GPEs for ESDs have developed rapidly (Fig. 1a). Figure 1b highlights the development of representative lignocellulosic gel polymer electrolytes. To date, most of the research has been focused on discovering novel gel electrolytes, as they play a crucial bridging role in energy storage devices. More impressively, it is also clear that the exponential rise in publications on gel electrolytes demonstrates the great enthusiasm of the scientific community in this field (Fig. 1c).

In the existing literature, there is no comprehensive review addressing the strategy for the design and engineering of lignocellulose-mediated gel polymer electrolytes for advanced energy storage devices. Most of the relevant reviews focus on biomass-based electrolyte preparation methods or single energy storage device applications (e.g., for lithium-ion batteries only). Therefore, we reveal the differential regulation strategies of lignocellulosic matrix elements (mechanical enhancement of cellulose, interfacial stabilization of lignin, and ionic solvation of hemicellulose) by resolving the common problems (e.g., ionic mobility kinetics) and specific bottlenecks (e.g., zinc dendrimer inhibition) faced by L-GPEs in different devices; and, at the same time, we emphasize the art of balancing its sustainability and high performance to provide a green energy storage. At the same time, we emphasize the art of balancing sustainability and high performance, and provide guidance for the design of green energy storage electrolytes from “molecular tailoring” to “device integration.” In this review, we have reviewed the key properties (*e.g*., ionic conductivity, interfacial compatibility, mechanical properties, and thermal stability) and design strategies of lignocellulosic gel electrolytes and established the connection between different performance characteristics and functional design approaches. Meanwhile, the applications of lignocellulose gel electrolytes in lithium-ion batteries, sodium-ion batteries, zinc-ion batteries, supercapacitors, and solar cells are also highlighted. The development prospects and challenges of lignocellulose gel electrolytes for energy storage are comprehensively discussed. It is hoped that the study in this review will be useful for future research on lignocellulose-based applications in the field of energy storage.

## Main Challenges of GPEs for Energy Storage

**Fig. 2:**
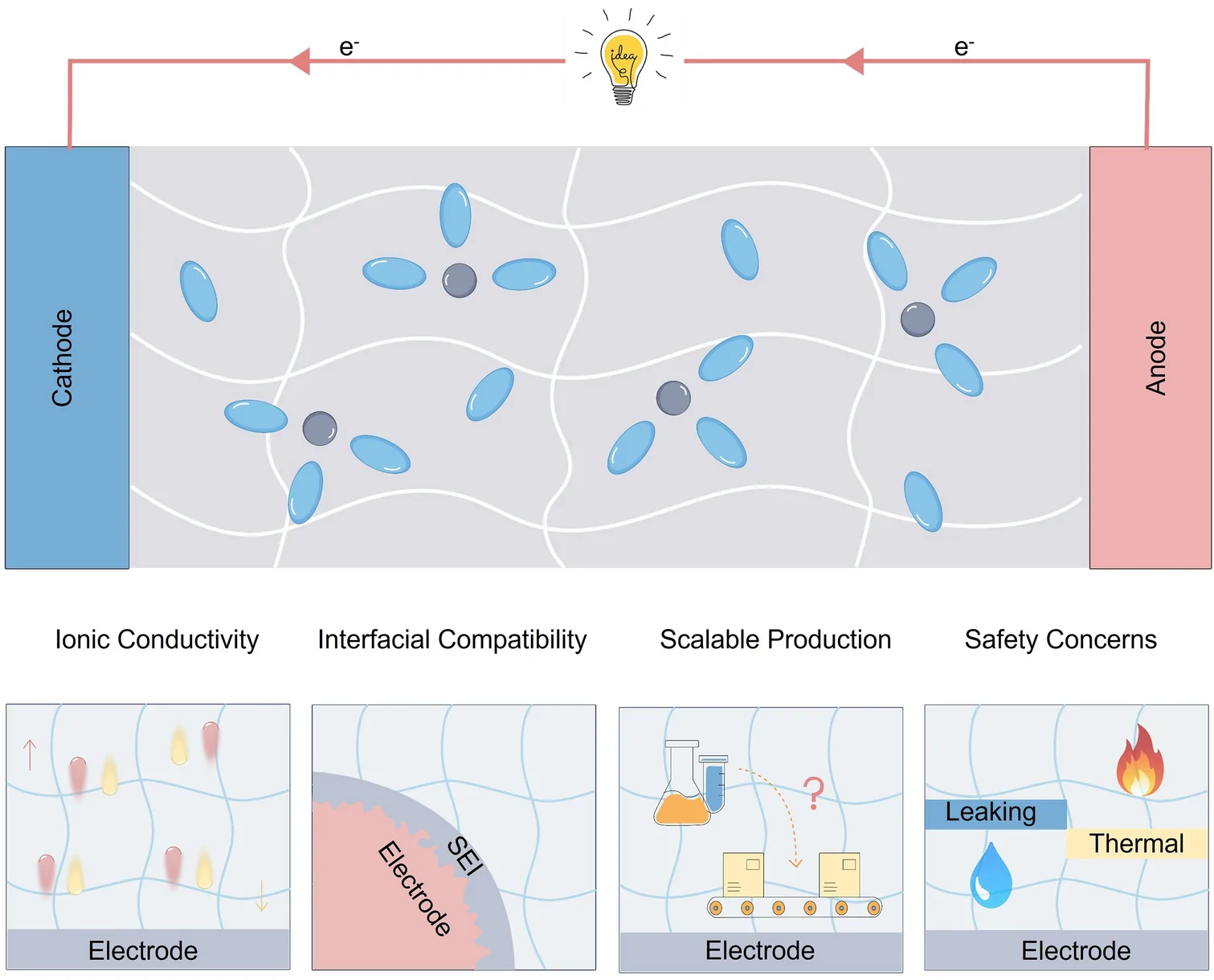
Main challenge of GPEs for energy storage

The demand for efficient and reliable energy storage devices is at an all-time high in the era of rapid development of modern society [43, 44]. From portable electronic devices to large-scale power grids, energy storage technologies play a crucial role in ensuring a stable supply of energy and promoting the utilization of renewable energy [45, 46]. Among various energy storage materials, gel polymer electrolytes (GPEs) have attracted widespread attention due to their unique properties, such as high ionic conductivity, good flexibility, and good interfacial compatibility. However, despite their great potential, GPEs face a series of major challenges in the application of energy storage devices. These challenges not only limit the performance and reliability of GPE-based energy storage devices but also pose barriers to their large-scale commercialization. In the next sections, we will delve into the major challenges of GPE for energy storage devices, which are essential to guide research efforts and drive the development of next-generation energy storage solutions (Fig. 2).

### Ionic Conductivity

One of the most significant challenges in the research of GPEs is their low ionic conductivity compared to liquid electrolytes, which is crucial for the performance of energy storage devices such as lithium-ion batteries and supercapacitors. Ionic conductivity determines the rate at which ions can move through the electrolyte, affecting the overall efficiency and power density of the device [47]. In electrolytes, ionic conduction arises from the combined effects of chemical and electrochemical potential gradients. For dilute solutions, a reasonable assumption is that nearly all charge carriers are effectively available [48]; such that the conductivity can be characterized using the Kohlrausch summation principle:

1 $$\sigma =\sum_{i}{\mu }_{i}{q}_{i}{n}_{i}$$

where the variables σ, ${\mu }_{i}$, ${q}_{i}$, and ${n}_{i}$ are the conductivity, mobility, charge, concentration of the ion, diffusion coefficient, Boltzmann's constant, and Kelvin temperature, respectively. According to Eq. (1), ionic conductivity is influenced by both the mobility and concentration of charge carriers. In dilute electrolytic systems, it is reasonable to assume complete availability of all charge carriers, so that the parameter *n* in Eq. (1) directly corresponds to the stoichiometric ion count from dissociation. However, most polymer electrolytes are not dilute solutions. Therefore, Coulombic interactions among charge carriers play a key role in determining ionic conductivity. In highly concentrated electrolytes, the salts not only supply ions but also function as weak cross-linkers, causing the formation of ion pairs and ion clusters. Ion pairs cannot promote ionic conductivity because of the electroneutrality, and ion clusters are too large to move [49]. In addition, the formation of ion pairs or ion clusters hinders the migration of the polymer chains, decreasing the ionic conductivity and cation transfer number. Furthermore, GPEs, due to their solvent-containing nature, are highly sensitive to temperature and other environmental factors, which can lead to evaporation of the solvent and a consequent drop in conductivity. This sensitivity poses a significant challenge for their application under extreme conditions such as subzero temperatures, high heat, or mechanical deformation. In addition, the challenge of ionic conductivity of conventional GPEs varies significantly from one energy storage device to another. In LIBs, where Li⁺ has a small radius and high mobility activity, GPEs need to maintain high ionic conductivity at high voltage and have sufficient mechanical strength to inhibit lithium dendrite growth. However, the polar functional groups in the traditional polymer matrix are prone to strong coordination with Li⁺, which reduces the Li⁺ migration number, and the formation of ion-pairs is intensified under high voltage, which further hampers ionic conduction and decreases the conductivity [50]. For SIBs, the Na⁺ radius is large, and its migration is highly dependent on the pore structure of the GPEs as well as the movement of the polymer chain segments. The rigid network of conventional polymers restricts the movement of the chain segments, resulting in Na⁺ conductivity often below 10^–3^ S cm^−1^, which is difficult to meet the practical application requirements of SIBs, and even with less ion-pair formation, the conductivity is much lower than that in LIBs [51].

### Interfacial Compatibility

In addition to the above properties, the performance of the ESD is also affected by the interfacial compatibility between the GPE and the electrodes. Interfacial compatibility is typically described as chemical and electrochemical stability (thermodynamic stability), SEI/CEI layers formation (kinetic stability), and physical contact at the interface. In other words, interfacial compatibility may be described as interfacial stability and interfacial resistance. Conventional energy storage devices are always assembled by extruding the components of the electrode and electrolyte membranes, which causes the electrodes and electrolyte to simply slide, leading to an increased number of interfacial barriers. The poor contact between GPEs and electrolytes can cause large interfacial impedance. Basically, the large intrinsic impedance is due to the small bite force between the electrode and electrolyte. It will result in large polarization, poor rate capability, and a short lifespan. In zinc-ion batteries, zinc dendrites grow faster compared to lithium-ion batteries, which places extreme demands on the mechanical barrier properties of the GPE and the chemical stability of the interface, and requires simultaneous resolution of corrosion and dendrites to maintain the stability of the interface [52]. Supercapacitors rely on rapid ion adsorption/desorption at the interface between the electrodes and GPEs to realize energy storage, and their interfacial compatibility is mainly reflected in the high efficiency of rapid ion transport and interfacial charge transfer [53]. Conventional GPEs need to have a high specific surface area and uniform ion transfer channels to ensure that the ions at the interface can respond quickly during high-frequency charging and discharging, reducing the charge transfer resistance and maintaining stable capacitance performance.

### Scalable Production and Safety Concerns

The need for scalable production of GPE-incorporated flexible energy devices poses strict requirements for performance metrics. The scalability of GPE production is a challenge due to the complex synthesis processes and the need for precise control over the polymer matrix and ionic conductor to ensure consistent performance. Besides, functional GPEs are still limited to the lab level and are far away from real applications. On the other hand, thermal stability is a critical concern for energy storage systems. Because of the leakage and flammability of the liquid electrolytes, as well as the poor thermostability of traditional commercial polyolefin separators, the ESDs safety becomes a key issue. Adopting GPEs is an efficient strategy to prevent the leakage of LEs and use polyolefin separators. However, internal micro-shorting phenomena can lead to localized temperature increases, and the GPE matrix is unable to withstand the heat it generates, leading to shrinkage or thermal decomposition, which can result in direct contact or short-circuiting between the electrodes.

In conclusion, while GPEs offer promising advantages for energy storage devices, significant challenges remain in enhancing their ionic conductivity, interfacial compatibility, production, and safety performance. To address these challenges, the development of advanced electrolytes is imperative to mitigate the above-mentioned drawbacks and achieve properties including efficient ionic conduction and mass transport, superior chemical/electrochemical stability against electrodes, and adequate thermal/mechanical tolerance. However, with the continuous progress of materials science, the emergence of new energy storage materials provides new opportunities to break through these technical bottlenecks. Among them, lignocellulose has gradually become a research hotspot in the field of energy storage due to its unique physicochemical properties and sustainability advantages. In the following sections, the advantages and applications of lignocellulose in energy storage will be elaborated in detail, to provide new perspectives and methodological basis for solving the problems of GPEs.

## Opportunities and Superiority of Lignocellulose for Constructing GPEs

### Source and Constituents

Lignocellulose, a compound with organic and inorganic components, is currently seen as a potentially renewable resource for the preparation of high value-added products [38, 54]. Lignocellulose is one of the most abundant biomass resources in nature and is widely found in plant cell walls [55, 56]. Its main sources were included as follows. Firstly, wood is the predominant source of lignocellulose and can be divided into hardwoods (*e.g*., angiosperms) and softwoods (*e.g*., gymnosperms) [57]. Notably, hardwoods are denser and grow more slowly than softwoods. Hardwoods are usually made up of 40%-55% cellulose, 24%-40% hemicellulose, and 18%-25% lignin, while softwoods are made up of 40%-45% cellulose, 25%-35% hemicellulose, and 25%-35% lignin [58]. Secondly, lignocellulose can be abundantly obtained from agricultural waste, which is inexpensive and easily accessible [59]. Corn straw, the world's most productive cereal, has an estimated annual global production of 1 billion tons, including 250 million tons in the USA and 220 million tons in China [60]. Its high carbohydrate content allows for the production of biofuels such as ethanol [61]. Sugarcane is a source of lignocellulosic biomass with the production of about 279 million tons of bagasse. Among them, Brazil is one of the largest producers with about 739.3 million tons per annum, which is followed by other large countries such as India, China, and the USA [62]. Besides that, rice production is dominated by Asian countries. The annual global straw production is 731 million tons, of which 667.7 million tons come from Asia [63]. The production rate of rice straw is 1–1.5 kg kg^−1^ of transplanted rice, where the cellulose content of this straw ranges from 33 to 38%. Finally, perennial forages (such as miscanthus and switchgrass), as herbaceous plants that grow continuously for many years without needing to be replanted annually, are also an important source of lignocellulose due to their long-term stable supply, high biomass yield, renewability, and adaptability. Among them, Manzanita as a promising lignocellulosic feedstock is characterized by high biomass yield, wide climatic diversity, and low chemical inputs. Annual yields of 5.5–36 tons ha^−1^ have been reported for manzanita, while yields of 8–44.1 tons ha^−1^ have been reported for willowherb [64].

**Fig. 3:**
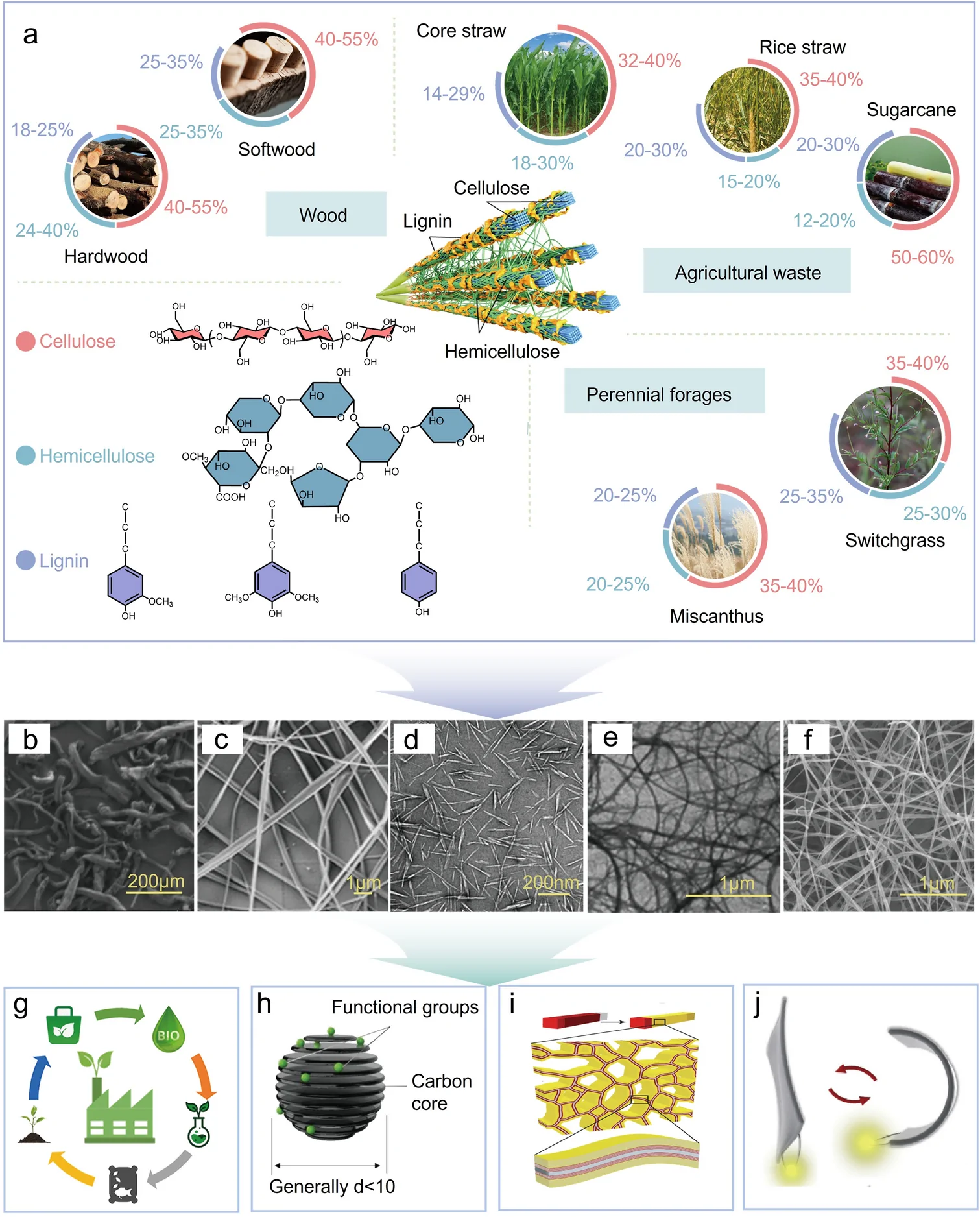
Overview of lignocellulose presentations. **a** Sources and constituents of lignocellulosic materials; The morphology of representative cellulose derivative and nanocellulose, including **b** carboxymethyl cellulose (CMC), **c** cellulose acetate (CA), **d** cellulose nanocrystals (CNCs), **e** cellulose nanofibers (CNFs) and **f** bacterial cellulose (BC); Various properties of lignocellulosic materials, including **g** renewability, **h** high carbon content, **i** porous structure and **j** mechanical properties

Lignocellulose is typically made up of three major constituents: cellulose, hemicellulose, and lignin (Fig. 3a) [65, 66]. Cellulose is the center of the lignocellulosic matrix, with lignin and hemicellulose acting as binders and fillers to encapsulate the cellulose molecules into protofibrils or protofibril bundles, which are attached by chemical and physical bonding [67, 68]. Cellulose is a polysaccharide in the form of a linear chain of repeating anhydrous D-glucose units covalently linked by *β*-1,4-glycosidic bonds [69, 70]. From the molecular structure of cellulose, cellulose exists in the form of linear chains which are bonded to each other by hydrogen bonds to form microfibers [70]. The microfibrils further aggregate to form larger structures called fiber bundles [71]. These microphonic fibers, together with hemicellulose and lignin, constitute the cell wall, which unites with other components (such as proteins and inorganic compounds) to ultimately form the plant [72]. Various types of cellulose material (including carboxymethyl cellulose (CMC) [73], cellulose acetate (CA) [74, 75], cellulose nanofibers (CNFs) [76–78], bacterial cellulose (BC) [79, 80], and cellulose nanocrystals (CNCs) [81–83]) can be obtained by mechanical treatment, enzymatic hydrolysis and acid hydrolysis (Fig. 3b–f). CNCs are rigid rod-like particles with diameters between 5 and 50 nm and lengths between 100 and 500 nm, whereas CNFs have diameters of less than 100 nm, lengths of a few micrometers, and large aspect ratios [76, 84, 85]. Cellulose is characterized by high crystallinity and dense hydroxyl network, which can be used for gel electrolyte mechanical enhancement and ion channel construction. And its rigid chains can be cross-linked by hydrogen bonding to form a 3D network structure with strong tensile strength, which can effectively inhibit dendrite penetration. Meanwhile, its hydrophilic group can promote electrolyte infiltration. In addition, cellulose-derived materials (e.g., CNFs) have nanoscale pores, which can provide low tortuosity transport paths for ions and further improve ionic conductivity [65].

Unlike cellulose, hemicellulose is a polysaccharide composed of the compounds d-glucose, d-xylose, d-mannose, d-galactose, d-galacturonic acid, d-arabinose, 4-O-methyl glucuronic acid, and d-glucuronic acid [86]. Similar to cellulose, these sugars are linked by *β*-1,4 or *β*-1,3 glycosidic bonds and the main chain can form a branched structure at positions (1 → 2), (1 → 3), or (1 → 6) [87]. This complex mixed system of multiple molecules, combined with the random nature of the short-chain structure, leads to a more complex separation process, which limits the potential of such polymers for use in related applications. Compared to the rigid structure of cellulose, hemicellulose, with its amorphous region and highly branched character, allows for interfacial capacitation and flexible regulation. Its abundant free hydroxyl groups can act as dynamic cross-linking sites, which can further enhance the cycling stability of the gel electrolyte through hydrogen bonding reorganization.

Lignin is a three-dimensional (3D) amorphous heteropolymer that typically consists of three major phenolic units, namely syringyl (S), guaiacyl (G), and hydroxyphenyl (H) units [88, 89]. The structural complexity of lignin mainly derives from the fact that its monomeric units are connected by two main categories of linkages, namely ether linkages such as *α*-O-4 and *β*-O-4 and carbon–carbon couplings including *β*-*β*, *β*-5, 5–5, and *β*-1, forming a highly branched three-dimensional network [90]. Unlike cellulose, lignin is an amorphous polymer that lacks a regular crystal structure and is mainly found in plant cell walls [39, 91]. Lignin binds to cellulose and hemicellulose through ether bonds, and in particular to hemicellulose through ester bonds, forming a rigid structure [92]. It plays a supportive and protective role in the plant and also influences growth, development, and resistance. Lignin can confer electrochemical protection to GPEs through the reactivity of the aromatic rigid backbone with phenolic hydroxyl groups. Its hydrophobic benzene ring can even be oriented at the electrode interface, forming a mechanical barrier to inhibit dendrite growth.

### Structures and Properties

Lignocellulose provides the basis for its global abundance, renewability, and consistency with the principles of a circular bioeconomy as an energy storage material (Fig. 3g) [72, 93]. Lignocellulose is the most abundant biomass resource in nature and is widely distributed in a variety of plants, such as agricultural wastes (wheat straw, rice straw), forestry by-products (sawdust, bark), and energy crops [94]. Its wide range of sources and renewability make lignocellulose a sustainable feedstock that can meet the growing demand for energy storage while reducing dependence on finite fossil resources [95]. In addition, the sustainability of lignocellulose is reflected in its carbon–neutral character. Unlike fossil fuels, lignocellulose absorbs carbon dioxide during growth, and the carbon dioxide released during use and decomposition does not add to the carbon content of the atmosphere [96]. This helps mitigate climate change and is in line with the global trend of transitioning to a low-carbon economy. In terms of energy storage, lignocellulosic materials not only have good chemical stability and mechanical properties but also significantly increase the capacity, energy density, and cycle life of energy storage devices. Therefore, lignocellulose, as a sustainable and richly sourced material, has great potential in the field of energy storage and offers the possibility of realizing green and efficient energy storage solutions.

Lignocellulose is mainly composed of cellulose, hemicellulose, and lignin, which can be converted into materials with high carbon content during high-temperature carbonization (Fig. 3h) [97, 98]. The high carbon content of lignocellulose gives it good electrical conductivity in energy storage devices, which can effectively facilitate the transmission and storage of electrical charges [38, 99]. The electrical conductivity of lignocellulose-based carbon materials can be further improved by various methods, such as high-temperature carbonization, chemical activation, and composite material preparation [100]. Kuzmenko and coworkers prepared cellulose materials from cellulose acetate by electrostatic spinning and acetylation and then carbonized the cellulose materials to obtain nitrogen-doped carbon nanofibers [101]. Studies have shown that high nitrogen doping has an impact on both its electrochemical properties and electrical conductivity. Therefore, when comparing undoped carbon nanofibers with lower electrical conductivity to n-doped carbon nanofibers, the material with higher electrical conductivity exhibits the best performance.

From the perspective of intrinsic microstructure, the porous structure and high specific surface area of lignocellulosic materials are key advantages for their application in energy storage devices (Fig. 3i) [44, 102]. The hierarchical porous structure of lignocellulose provides abundant ion transport channels for its use as a gel electrolyte [103]. This porous structure not only improves the ionic conductivity of the electrolyte but also facilitates the contact between the electrolyte and the electrodes, thus increasing the speed and efficiency of the electrochemical reaction. For example, Qiu and coworkers synthesized lignocellulose (LC) membranes with high porosity using a simple solution-casting method [104]. The results showed that the ionic conductivity of LC-GPE was 32.6 mS cm^−1^ at 25 °C. The ultra-high ionic conductivity was mainly due to its high porosity (86.2%) and excellent electrolyte absorption (948 wt%). The high porosity not only allows more space for electrolyte storage but also provides channels for ion transport. The excellent electrolyte absorption not only enhances the number of carriers but also reduces the ion migration resistance due to the liquid electrolyte-like fluidity of GPE. In addition, the nanofiber structure of lignocellulose can form a stable three-dimensional network [105], which enhances the structural stability of the gel electrolyte and prevents deformation or rupture during charging and discharging.

Lignocellulosic materials exhibit multiple advantages in energy storage devices that are closely related to their unique mechanical properties, intrinsic microstructure, and chemical activity [106]. Firstly, in terms of mechanical properties, lignocellulosic materials, with their natural fiber structure and adjustable physical properties, give the materials excellent tensile strength and toughness, enabling them to withstand all kinds of mechanical stresses that may be encountered during the operation of the energy storage device, and also enabling them to be processed into a variety of shapes and sizes, to meet the design needs of different energy storage devices (Fig. 3j) [107]. For example, Zhao and coworkers prepared BC/PVA hydrogel electrolytes with superior mechanical properties using bacterial cellulose [108]. Due to the hydrogen bonding interactions between the PVA matrix and the BC microfibers, the mechanical strength of these hydrogel membranes is enhanced with the increase of BC content and can form percolating dual networks. Moreover, this membrane can be made into various shapes by arbitrary folding and can be restored to its original size, a feature that reflects its superior flexibility. In addition, lignocellulosic materials have low densities, typically in the range of 1.0–1.5 g cm^−3^, which allows them to significantly reduce the overall weight of energy storage devices. This excellent mechanical property allows lignocellulose-based gel electrolytes to have a wide range of potential applications in flexible energy storage devices, capable of maintaining structural integrity and functional stability when subjected to external forces.

## Key Requirements and Engineering Strategies of L-GPE for Energy Storage

**Fig. 4:**
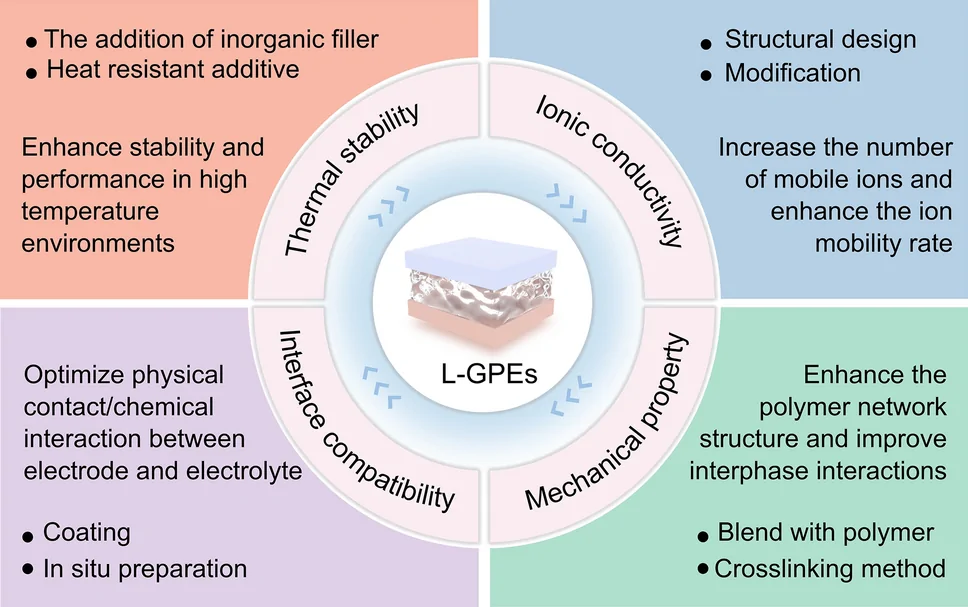
Schematic diagram of key requirements and engineering strategies of L-GPE for energy storage devices

As discussed above, lignocellulose, as an abundant renewable resource, has received increasing attention for its potential applications in energy storage [109, 110]. Its unique chemical structure and sustainability features make it an ideal candidate for the development of high-performance gel electrolytes. However, the successful application of lignocellulose in energy storage devices needs to fulfill a number of key requirements, including high ionic conductivity, excellent mechanical strength, superior electrochemical stability, and compatibility with electrode materials (Table 1). In this section, based on an overview of the properties of lignocellulose and existing research, the key requirements and engineering strategies for lignocellulose-mediated gel electrolytes in energy storage devices will be further explored (Fig. 4).

**Table 1:** Summary of L-GPEs for ESD

L-GPEs	Strategies	Electrolyte uptake (%)	Tensile strength (MPa)	Flame retardance	Ionic conductivity (mS cm^−1^)	Cycling performance	Application	Refs
CNF/PEG	Structural design	–	0.75 ± 0.09	Yes	0.61 ± 0.12	94% capacity retention after 300 cycles at 1 C	Lithium-ion batteries	[28]
Cellulosic Zn-gel	Structural design	–	0.88	–	8.39	~ 90% efficiency after 500 cycles at 0.5 C	Zn-ion batteries	[111]
MC/AC	Modification	628.5	25	–	4.36	91.6% capacity retention after 100 cycles	Lithium-ion batteries	[112]
Cellulosic poly (PILs)/PVA	Modification	–	0.19	–	4.2	90% capacity retention after 8000 cycles	Supercapacitors	[113]
HEC/LC	Blending	425	6.3	–	2.68	92% capacity retention after 100 cycles	Lithium-ion batteries	[114]
Chitosan/lignocellulose	Blending	749.1	44.0	–	2.89	98.5% capacity retention after 100 cycles	Lithium-ion batteries	[115]
Cellulose/PVA	Crossing	–	0.30	–	105	70% capacity retention after 2500 cycles	Lithium-ion battery	[116]
LCNF	–	–	0.1	–	10	100% capacity retention after 3000 cycles	Supercapacitors	[117]
LC	Modification	144	4.62	–	2.42	408 mAh g^−1^ after 50 cycles	Lithium sulfur batteries	[118]
SA/LC	Blending	337	5.04	–	2.7	–	Lithium-ion batteries	[119]
PEG/LC	Blending	267	4.42	–	3.22	99.4% capacity retention after 50 cycles at 0.2 C	Lithium-ion batteries	[120]
Potato starch/LC	Blending	194	10.65	–	1.27	–	Lithium-ion batteries	[121]
PEG/cellulose	Crossing	236	211.06	–	3.31	–	Lithium batteries	[122]
HK/CMC	Blending	–	58	–	24.56	98.02% coulomb efficiency after 2000 h	Zinc-ion batteries	[123]
Cellulose	Crossing	–	4.42	–	38.6	99.4% capacity retention after 2000 cycles	Zinc-metal batteries	[124]
CMC	–	-	1.33	–	34.5	99.54% coulomb efficiency after 500 cycles	Zinc-ion batteries	[125]
CMNC	Crossing	312	67.45	–	3.93	98% coulomb efficiency after 50 cycles	Lithium-ion batteries	[126]
PETEA/CAP	In situ	–	–	–	1.03	90.6% capacity retention after 500 cycles	Lithium metal batteries	[127]
Cellulose/PEGDA	Crossing	932.57	0.30	–	4.60	96/9% capacity retention after 100 cycles at 0.5 C	Lithium-ion batteries	[128]
QACA	Coating	–	1.74	–	1.05	383.9 mAh g^−1^ reversible capacity after 100 cycles	Lithium–sulfur batteries	[129]
PVDF HFP/cellulose	Coating	526.11	7.6	Yes	1.47	91.7% capacity retention after 500 cycles	Lithium-ion batteries	[130]
PVDF-HFP/CA	Heat resistant additive	–	–	Yes	1.08	93% capacity retention after 600 cycles	Lithium metal batteries	[131]
CA/PEGDA/BN	Inorganic filler	–	–	–	8.9	77% capacity retention after 200 cycles	Lithium-ion batteries	[132]

### Ionic Conductivity

The ionic conductivity, as a key parameter in electrolyte research work, directly determines whether the electrolyte can be operated to achieve an effective transfer of the theoretical energy density of the energy storage device system. It is of great importance to establish an ionic conductivity threshold for a wide range of electrolyte systems in order to guide electrolyte optimization with the aim of achieving performance enhancement for a wider range of applications. High ionic conductivity not only improves the charging and discharging rate of the ESDs but also enhances the power density of the ESDs, thus improving the overall performance. The activation energy, as a core parameter characterizing the kinetic barriers to ion migration in gel electrolytes (GPEs), maps directly to the rate of ionic conduction processes-low activation energy tends to correspond to fast ionic transport kinetics, which is then reflected in high ionic conductivity. Reducing the activation energy is usually achieved by increasing the cation concentration, optimizing the cation migration rate, and constructing multiple cation conduction channels. Given that GPEs combine the cohesive properties of a solid matrix with the diffusive capabilities of a liquid electrolyte, the composition of the GPE, including the polymer matrix and encapsulated liquid phase, needs to be synergistically modulated to achieve a performance coupling of high ionic conductivity and low activation energy. For L-GPE, its ionic conductivity is affected by a variety of factors, including the chemical structure of the polymer matrix, the type and concentration of electrolyte salts, the use of plasticizers, and the structural design of the electrolyte. To improve the ionic conductivity of L-GPE, researchers have adopted various strategies.

#### Structural Design

**Fig. 5:**
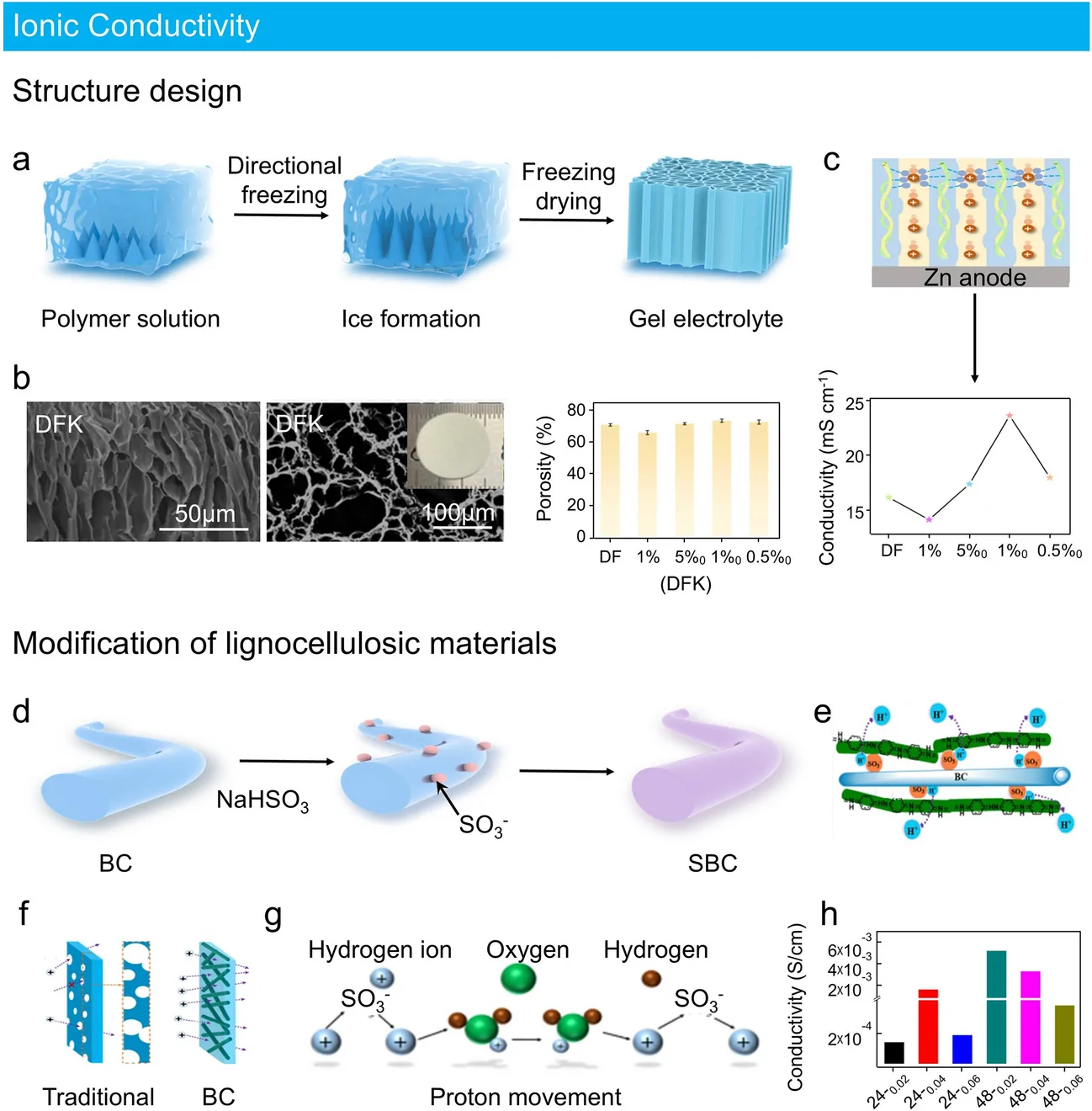
Engineering strategies for improving ionic conductivity of L-GPE. **a** Schematic illustration of gel electrolytes through structural design. **b** SEM image of DFK and Porosity of the DFK. **c** Schematic diagram of the ion transport mechanism in DFK electrolyte and ionic conductivity of DFK. (Reproduced with permission from Ref. [133], Copyright 2024, American Chemical Society). **d** Schematic illustrations of gel electrolytes through modification of LCs. **e** Schematic diagram of the protons transport in SBC/PANI GPEs. **f** Ion transport in traditional porous electrolyte and SBC/PANI GPEs. **g** Schematic diagram of the formation of the ‘Water Bridge’. **h** Ionic conductivity of SBC/PANI membranes. (Reproduced with permission from Ref. [134], Copyright 2017, Elsevier)

The goal of the structural design is to ultimately achieve high ionic conductivity and low interfacial impedance by constructing a continuous, low tortuosity ion transport network while inhibiting polymer crystallization and enhancing electrolyte electrode interfacial contact. With the advantages of a high aspect ratio and high modulus, CNFs can ensure a mechanically strong network even at very low concentrations [135]. In addition, the porous structure of the CNFs network facilitates the formation of abundant ion migration pathways, which allows for rapid ion transport and thus improves the charging and discharging efficiency and power density of the energy storage device. Wang and coworkers utilized the high aspect ratio and undried state of CNFs to prepare high-performance LIBs electrolytes by dissolving them in different concentrations of NaCl solution and modulating the network pore structure [136]. Swelling the CNF network in salt solutions of varying concentrations induces its expansion into a more disordered orientation and promotes the opening of z-axis-aligned pores, which further regulates the porosity and fiber orientation of the CNF network and thus the ionic conductivity of the CNF/PEG electrolyte. The CNF dynamic network increased the ionic conductivity compared to the polyethylene glycol electrolyte. The strategy resulted in a room temperature ionic conductivity of 0.61 ± 0.12 mS cm^−1^ for the electrolyte, which is one of the highest among polymer gel electrolytes. Cellulose-based gels, with controllable nanostructures, strong mechanical strength, and tunable ionic conductivity, show great potential for applications in the field of energy storage devices, but have encountered challenges in scaling up. Therefore, to achieve molecular-scale fabrication of cellulose-based gels, Zheng and coworkers developed an innovative cellulosic Zn-gel by the deep eutectic solvent (DES) method [111]. Unlike the conventional liquid immersion approach, this technique introduces ethanol vapor to precisely modulate the assembly behavior of cellulose molecular chains, resulting in the formation of cellulosic Zn-gel characterized by tightly interconnected molecular networks. The self-assembly of cellulose molecules is induced by ethanol vapor, and the entropy-driven ethanol molecules gradually penetrate the cellulose system, and at the same time guide the orderly arrangement of cellulose molecular chains through hydrogen bonding to form a densely interconnected molecular topological network. This ordered structure reduces the “path bending” of ion transport and lowers the transport resistance; while the dense hydrogen bonding network both enhances the mechanical stability of the gel and provides continuous “hydrogen bonding-mediated transport channels for Zn^2^⁺,” which promotes the directional migration of ions. Interestingly, cellulosic Zn-gel treated with ethanol for 6 h reached peak ionic conductivities of up to 8.39 mS cm^−1^, exceeding the ionic conductivity levels of many previously reported cellulosic Zn-gel. The development of such self-supporting cellulosic Zn-gel is an important step forward, providing a green, scalable, and efficient pathway for the fabrication and widespread adoption of flexible Zn-ion batteries (FZIBs). To further enhance the ion transport path, it has been investigated to construct vertically aligned pore structures using the ice template method. Ji and coworkers prepared a bi-porous gel electrolyte by directional freeze-drying (Fig. 5a) [133]. Through the directional freeze-drying technique, DFK formed vertically aligned directional channels, which significantly shortened the ion migration paths and reduced the transport time. The secondary pores introduced by K₂CO₃ as a pore-forming agent were combined with the directional channels to form a hierarchical structure of “main channel–branching pores,” which provided additional transport paths with a low degree of curvature and reduced the ions in the scattering and retention of ions in transport (Fig. 5b). The porosity of the resulting polymer membrane (DFK) was as high as 73.4%. Its two-pore structure also effectively redistributes the Zn^2+^ ionic flow and promotes the uniform deposition of Zn^2+^. As a result, the DFK achieved impressive electrolyte uptake (1777%), ionic conductivity (23.6 mS cm^−1^), and mechanical strength (2.0 MPa) (Fig. 5c). The DFK electrolyte exhibits excellent cycling stability and multiplicity performance in K/Zn bionic batteries. Its discharge specific capacity reaches 0.068 mAh cm^−2^ (68.0 mAh g^−1^) at a current density of 1 mA cm^−2^, and maintains 85.3% capacity retention after 1000 cycles. In addition, it has a high energy density (134.5 Wh kg^−1^) and power density (5.2 kW kg^−1^).

#### Modification of Lignocellulosic Materials

The chemical structure and physical morphology of lignocellulose have a decisive influence on the ion transport properties of L-GPE. Through chemical modification at the molecular level, nano-scale structural engineering, and multi-component composite design, the ion affinity, charge density and transport channel continuity of cellulose can be significantly optimized to break through the bottleneck of conductivity of traditional electrolytes (Fig. 5d). Zhu and coworkers prepared gel electrolytes by modifying cellulose and then compounding it with a polyvinyl alcohol base, taking advantage of the cellulose superbase/DMSO/CO_2_ cellulose solvent system [113]. Linear cellulose polyethers (PILs) can be prepared in situ by introducing cyclic anhydride in a superbase /CO₂/DMSO solvent system for cellulose. The PILs provided a high concentration of free ions, and their small ionic radius reduced the migration resistance and enhanced the conductivity. The results showed that the gel electrolyte constructed by this system exhibited a high ionic conductivity of 4.2 mS cm^−1^ at room temperature. The presence of allyl groups ensures mechanical strength during UV cross-linking, reduces the crystallinity of cellulose, and improves the flexibility of cellulose chains. Therefore, Yu and coworkers modified cellulose with chloropropylene to introduce allyl groups [112]. Polar functional groups such as hydroxyl groups in lignocellulose immobilize lithium salt anions (e.g., TFSI-) through hydrogen bonding and promote Li⁺ migration. The results showed that the GPE had a high Li^+^ transfer number (0.902) and ionic conductivity (4.36 × 10^–3^ S cm^−1^). However, too high AC content (e.g., 5.5% ACMC) leads to a decrease in ionic conductivity and mobility number due to an increase in crosslink density, indicating that the content of lignocellulose needs to be precisely regulated to balance the performance. There have been studies reporting that the introduction of sulfonic acid groups in materials can improve the ionic conductivity of composites used in fuel cells. Therefore, Yue and coworkers sulfonated BC with sodium periodate and sodium bisulfite [134]. Then SBC/PANI composite gel polymer electrolyte membranes were prepared using SBC nanofibers as templates. Since the introduction of sulfonic acid groups in BC can provide a large number of protons (Fig. 5e), these protons can move freely in the three-dimensional network of the SBC/PANI membrane. The BC-based gel electrolyte has a good three-dimensional network structure, which facilitates the migration of ions inside (Fig. 5f). According to the Grotthuss mechanism when a water molecule binds to a sulfonic acid group to form a hydrated hydrogen ion, the proton it carries will be transferred to a water molecule adsorbed by a neighboring sulfonic acid group, thus forming a ‘water bridge’ transfer structure. The smaller the water molecule spacing, the stronger the hydrogen bonding effect, and the faster the proton migration rate across the membrane (Fig. 5g). For SBC/PANI composite GPE, the ionic conductivity and ion exchange capacity were closely related to the degree of sulfonation (DS). The results showed that when the DS was 41.87%, the ionic conductivity and ion exchange capacity increased to 5.2 × 10^–3^ S cm^−1^ and 3.92 mequiv (Fig. 5h).

### Mechanical Properties

Mechanical properties of L-GPE are crucial for the performance and safety of energy storage devices. Firstly, excellent mechanical strength and toughness ensure that the electrolyte is able to withstand mechanical stress and deformation during cyclic charging and discharging, thus prolonging the life of the device. Second, appropriate modulus of elasticity and tensile resistance help maintain the structural integrity of the electrolyte under various operating conditions, preventing electrolyte rupture or delamination due to mechanical damage. In addition, good self-healing ability is also an important part of the mechanical properties, which can spontaneously repair the electrolyte after it has been subjected to localized damage, further enhancing its durability and reliability. To achieve these mechanical properties, research strategies often include blending with polymers or improving the overall mechanical stability of the gel through chemical or physical cross-linking. The combined application of these strategies not only enhances the mechanical properties of lignocellulose-mediated gel electrolytes but also lays a solid foundation for their wide application in high-performance energy storage devices.

#### Blend with Polymer

**Fig. 6:**
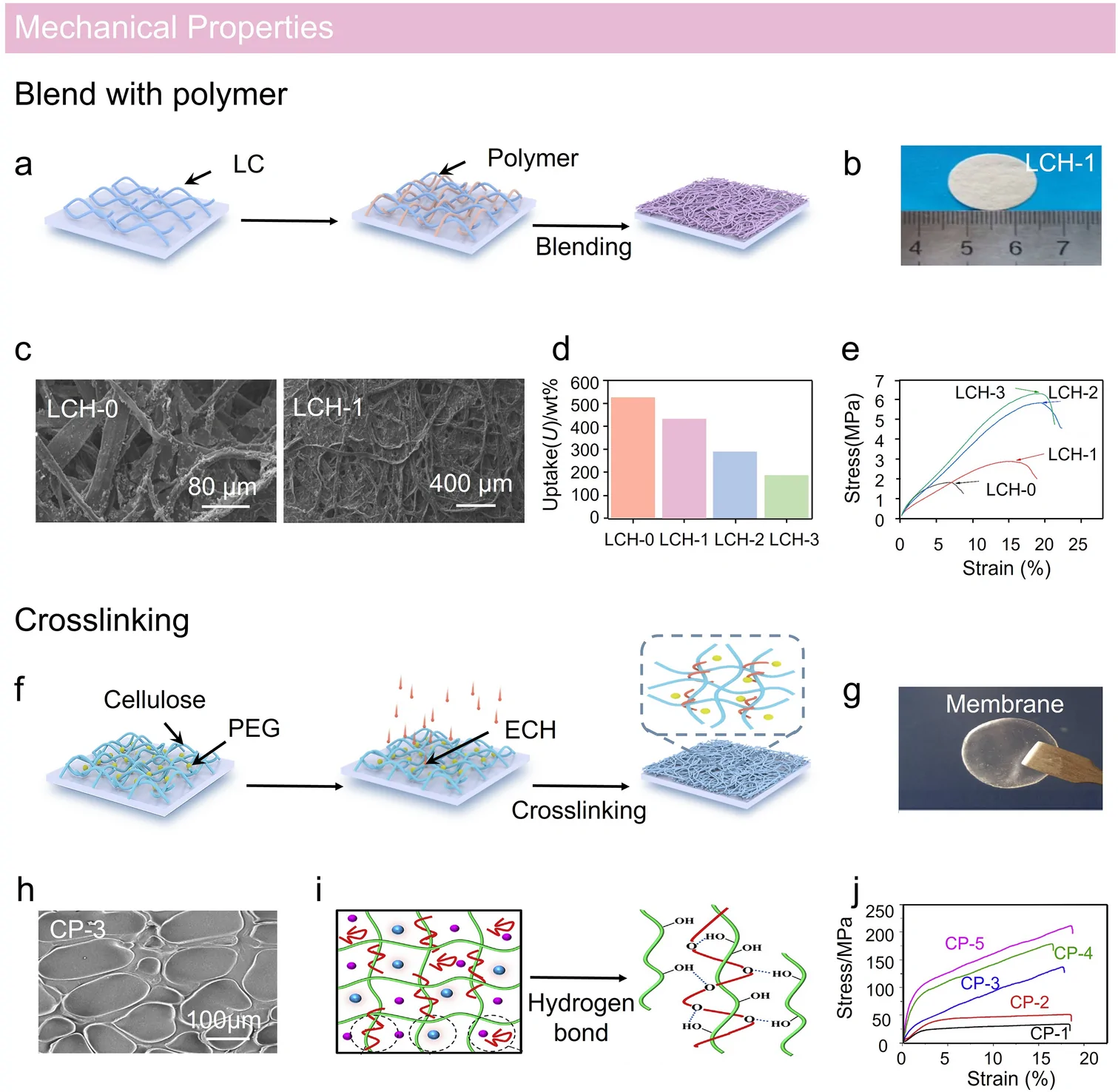
Engineering strategies for improving mechanical properties of L-GPE. **a** Schematic illustration of gel electrolytes through blending with polymer. **b** Diagram of the GPEs. **c** SEM image of LCH-0 and LCH-1. **d** Liquid electrolyte uptake of LCH membranes. **e** Stress–strain curves of GPEs (Reproduced with permission from Ref. [114], Copyright 2023, Indian Academy of Sciences). **f** Schematic illustrations of gel electrolytes through crossing method. **g** Diagram of the composite membranes. **h** SEM image of the composite membranes. **i** Schematic of the lithium-ion transporting mechanism of GPE. **j** Stress–strain curves of the composite membranes. (Reproduced with permission from Ref. [120], Copyright 2017, Elsevier)

Blending with polymer is one of the core strategies to enhance the mechanical properties of L-GPE by combining lignocellulosic derivatives (*e.g*. cellulose nanofibers, lignin) with synthetic or natural polymers to form a composite system with synergistic effects, thus overcoming the limitations of single materials (Fig. 6a). Hydroxyethyl cellulose (HEC), a cellulose derivative bearing abundant polar hydroxyl groups, effectively enhances the affinity between liquid electrolytes (LE) and polymer matrices. In this regard, Liu and coworkers prepared a gel polymer electrolyte (GPE) for lithium-ion batteries with better performance using hydroxyethyl cellulose (HEC) composite lignocellulose (LC) as the matrix (Fig. 6b) [114]. The viscosity and film-forming properties of HEC fill the natural pores of the LC (Fig. 6c), reducing structural defects and making the material more homogeneous. The liquid electrolyte uptake is a key parameter to ensure the ionic conductivity of GPE. As shown in Fig. 6d, the liquid electrolyte uptake decreased with the increase of HEC content. Notably, the dense structure inhibits crack extension and enhances tensile strength. The mechanical properties of the composite membrane were enhanced with the increase of HEC content, and the strength values of LCH-1 and LCH-2 were 2.89 and 5.85 MPa, respectively (Fig. 6e). Sodium alginate is rich in carboxyl groups, has good stability, and is not toxic. Therefore, Xu and coworkers prepared GPE by compounding LC with sodium alginate (SA) [119]. The results showed that the tensile strength and elongation at break increased with the increase of the SA-binding ratio. This is because potato starch forms a high-viscosity gel after pasteurization, which fills the pores between the LC fibers, forms a dense network structure, reduces defects, and enhances intermolecular forces. When the addition of SA reached 20 wt%, the mechanical strength of the composite membrane was more than four times that of the pure LC-based membrane. Potato starch has a high tensile strength due to its high molecular weight, which gives the starch paste a high viscosity. Song and coworkers selected LC and potato starch as matrix materials for GPE for LIBs [120]. The incorporation of starch significantly increased the tensile strength. It was shown that when the potato starch content was 5 wt%, the tensile strength of the composite membrane was 6 times higher than that of the pure LC membrane. This may come from two reasons: one is that the starch paste with high viscosity acts as a binder to strengthen the LC matrix, and the other is that the composite membrane may be gradually occupied by the starch component and show a denser structure. Although blending with polymers improves the mechanical properties, they all face the problem of conductivity attenuation, such as potato starch clogging the pores.

#### Crosslinking Method

Crosslinking is a key strategy to modulate the polymer network structure, and by introducing chemical or physical cross-linking points, the mechanical strength, elasticity and durability of L-GPE can be significantly enhanced while balancing the ion transport properties (Fig. 6f). The high strength and flexibility of PVA itself allow the gel electrolyte to adapt to the needs of flexible energy storage devices while maintaining structural stability. To prepare gel electrolytes with high mechanical properties, Zhang and coworkers prepared hydrogel electrolytes by directly mixing cellulose solution with PVA [116]. Hydrogen bonding will be formed between cellulose and PVA molecular chains, and this action will make the hydrogel structure more uniform and more dense, which is conducive to energy dissipation and improved mechanical properties. When the content of cellulose in the hydrogel is 5%, the composite hydrogel prepared (P-CE of 5%) has a tensile strength of 306 kPa (higher than that of pure PVA: 182 kPa) and an elongation at break of 1106%. Bacterial cellulose has a unique nanofiber network structure. These nanofibers have very small diameters, usually around a few tens of nanometers, and are interwoven to form a three-dimensional network structure with a high degree of crystallinity (crystallinity up to 60%-90%). This fine and dense network structure provides a strong supporting framework for the gel electrolyte, which can effectively disperse and withstand external forces, thus significantly improving its mechanical strength. In this regard, Zhao and coworkers prepared flexible hydrogel electrolytes (BPCE) by in situ synthesis of bacterial cellulose microfibrils (BC) and polyvinyl alcohol (PVA) [108]. The mechanical strength of the BPCE was increased by a factor of 9 (from 0.102 MPa for the original PVA to 0.951 MPa (6-BPCE) due to the presence of hydrogen bonds between the BC microfibrils and the PVA matrix, which formed a dual network for load-bearing permeation. Although the mechanical strength was improved to some extent, it was still low. Therefore, Zhang and coworkers prepared a double crosslinked (DC) cellulose hydrogel electrolyte (DCZ-gel) by a sequential chemical and physical crosslinking strategy [124]. Using epichlorohydrin (ECH) as the cross-linking agent, the epoxy group of ECH undergoes Williamson etherification and alkali-catalyzed oxyalkylation with the hydroxyl group (-OH) on the cellulose chain to form a covalently bonded cross-linked network in an alkaline LiOH / urea system. After chemical cross-linking, the hydrogels were treated with 75% ethanol, which induced the cellulose chains to form physical cross-linking domains through hydrogen bonding, chain entanglement, and cellulose type II crystalline hydrates. The rigid backbone of chemical cross-linking and the dynamic interactions of physical cross-linking form a “rigid-flexible” network. The unique DC network formed by this strategy resulted in a hydrogel electrolyte with excellent mechanical strength (2.08 MPa, 145%) and an abundant network of ion-transporting pores (38.6 mS cm^−1^). Carboxymethyl cellulose (CMC), as one of the cellulose derivatives, has good water solubility, film-forming, thickening, and stability. Its molecular chain contains a large number of carboxymethyl groups, which give CMC a strong hydrophilicity and enable it to dissolve rapidly in water to form a homogeneous solution. At the same time, the presence of carboxymethyl groups also gives CMC a certain ion exchange capacity, which is essential for the construction of electrolyte systems. However, the lack of mechanical properties due to the high degree of substitution of pure carboxymethylcellulose gel electrolytes inhibits the application of ZIBs for grid-scale storage. In this regard, Zhang and coworkers composite hydrolyzed wool proteins and carboxymethylcellulose to prepare gel electrolytes [123]. Hydrolyzed wool keratin is a small-molecule protein with amphiphilicity and abundant amino/carboxy groups. Under heating conditions, the protein and polysaccharide undergo a Maillard reaction, which can improve the mechanical properties of the CMC composite gel electrolyte. The results showed that the gel electrolyte had the maximum breaking strength (58 MPa) and maximum elongation at break (6.3%), which were higher than those of other electrolytes. The reason is that hydrolyzed wool keratin has excellent Young's modulus due to its abundant hydrogen, salt, and disulfide bonds, which makes the composite gel electrolyte compact and has good mechanical properties. In order to further improve the mechanical properties of GPE, Zhao and coworkers prepared cellulose/PEG composite GPE by a one-step cross-linking method (Fig. 6f) [122]. This GPE exhibits a porous and cross-linked network structure that ensures high liquid electrolyte uptake (Fig. 6g, h). The results showed that the tensile strength was significantly increased from 33.92 to 211.06 MPa after the addition of PEG (Fig. 6i, j). This was attributed to the formation of strong hydrogen bonding between hydroxyl groups in the main chain of cellulose and hydroxyl groups at the end of PEG. The interaction between polyethylene glycol and cellulose is the key factor to improve the mechanical strength. The cross-linking strategy significantly enhances the mechanical properties (strength, modulus, toughness) of L-GPEs through the formation of a three-dimensional network structure based on physicochemical principles such as restriction of chain movement, multiple energy dissipation mechanisms, and inhibition of solvent softening, which is a key advantage for its use as a safe electrolyte. However, major technical challenges such as balancing ionic conductivity and mechanical strength, ensuring excellent and stable electrode/electrolyte interfaces, developing efficient and reliable manufacturing processes, and guaranteeing long-term cycling stability must be overcome to successfully bring cross-linked L-GPEs to large-scale applications, as well as to simultaneously address the economic challenges such as high raw material costs, increased manufacturing costs, and supply chain maturity.

### Interfacial Compatibility

Interfacial compatibility refers to the degree of interaction and matching between the electrolyte and the electrode and is one of the key factors affecting the performance of energy storage devices. An ideal L-GPE needs to form a stable interface with the electrode at the chemical, mechanical, and electrochemical levels to avoid side reactions (*e.g*. electrolyte decomposition, lithium dendrite growth) and interface stripping. Good interfacial compatibility can reduce the resistance at the interface and improve the ion transport efficiency at the interface, thus enhancing the overall performance of the energy storage device. To achieve this goal, the engineering strategy can focus slightly on *in situ* polymerization versus coating approaches.

#### Coating

**Fig. 7:**
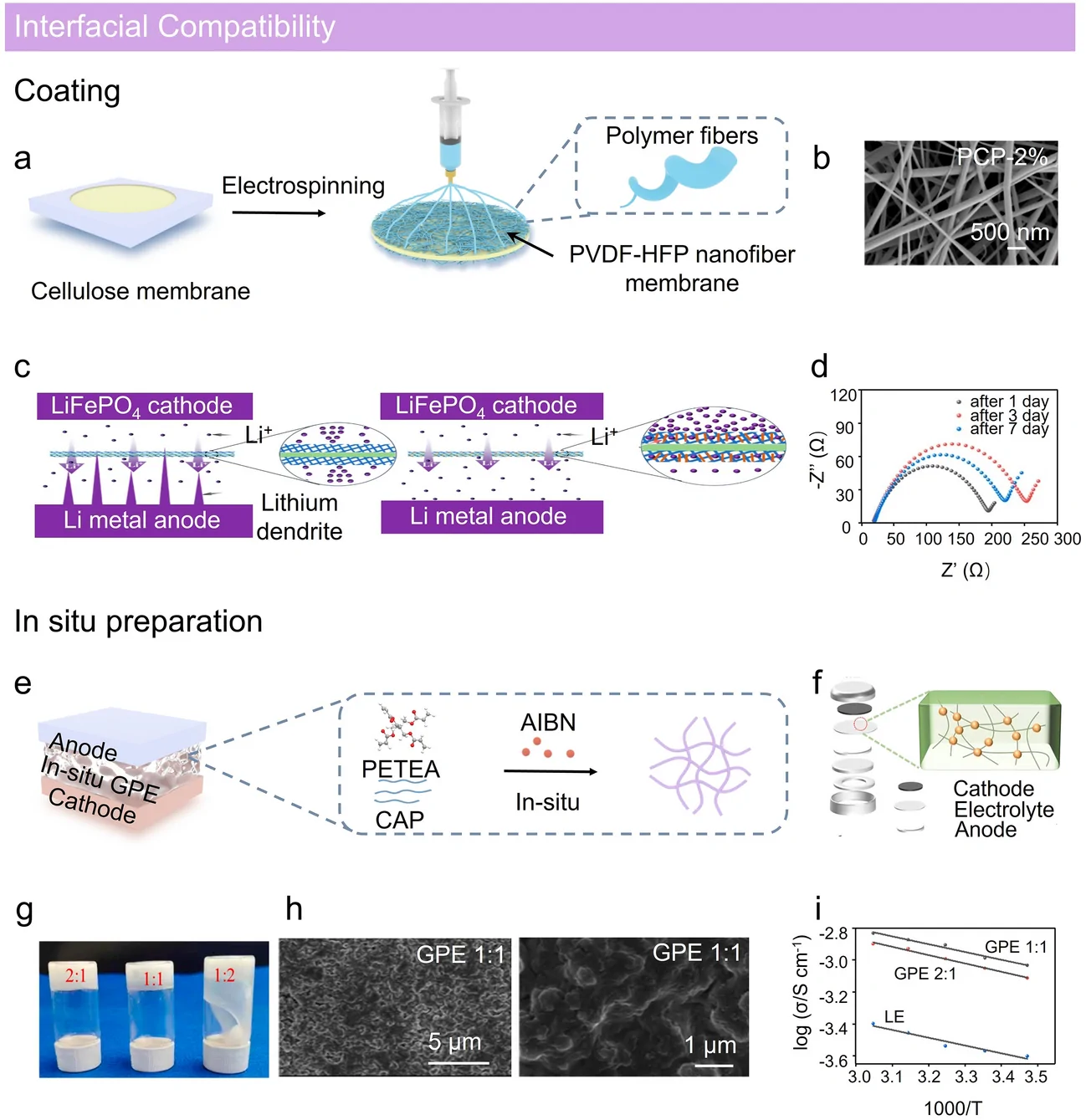
Engineering strategies for improving interfacial compatibility of L-GPE**. a** Schematic illustration of gel electrolytes through coating. **b** SEM image of PCPs. **c** Mechanism of nano-HT acts as a redistributor for lithium-ion transport. **d** EIS images of lithium symmetric batteries for PCPs. (Reproduced with permission from Ref. [130], Copyright 2024, ACS). **e** Schematic illustrations of gel electrolytes through *in-situ* preparation. **f** Schematic diagram of GPE. **g** Optical photographs of GPEs with different PETEA/CAP ratios. **h** SEM images of GPE. **i** Arrhenius plots of GPE 1:1, GPE 2:1, and LE. (Reproduced with permission from Ref. [127], Copyright 2023, Elsevier)

Coating technology optimizes the interface compatibility of the L-GPE by creating a functional protective layer on the electrode or electrolyte surface (Fig. 7a). The homogeneous and dense coating blocks direct contact between the electrolyte and the electrode and inhibits side reactions (*e.g*., electrolyte decomposition, violent reaction between lithium metal and electrolyte). In addition, the coating material can guide the uniform deposition of ions and avoid the growth of dendrites caused by high local current density. The hydroxyl groups on the surface of cellulose-based gel polymer electrolytes lead to poor interfacial compatibility due to side reactions with lithium wafers. Compared with polyvinylidene fluoride (PVDF), the C-F chemical bond in PVDF-HFP is characterized by a high dielectric constant, low surface energy, and excellent resistance to chemical degradation. These advantages make the PVDF-HFP membrane contact with the electrode without any side reaction and improve the interfacial compatibility. However, there are defects in its mechanical properties, and it is considered that adding nanoclay particles (*e.g*., hydrotalcite) to it can improve its mechanical properties. Zi and coworkers prepared a novel cellulose-based gel polymer electrolyte by uniformly coating nano-hydrotalcite/PVDF-HFP composites on the surface of cellulose membranes using electrostatic spinning technology (Fig. 7b) [130]. The cellulose-based gel polymer electrolyte has good interfacial compatibility, which is mainly reflected in the ability of Li^+^ to transfer from the electrode to the electrolyte (Fig. 7c). As shown in Fig. 7d, the interfacial resistance of PCP can reach a minimum value of 194.5 Ω, which is much lower than that of CM. This is because the nanohydrotalcite on the surface of the cellulose membrane can effectively prevent contact between hydroxyl groups and lithium flakes, reduce side reactions, and improve interfacial compatibility. In addition, nanohydrotalcite can act as a Li^+^ transport redistributor to promote the uniform deposition of Li^+^, reduce the formation of lithium dendrites, and extend the cycle life. In another study, Huang and coworkers designed a bifunctional asymmetric cellulose gel electrolyte [129]. The structure consisted of two parts, one part was a pure cellulose gel electrolyte (labeled as QACA) prepared from allyl-modified cellulose (AC) and cellulose acetate (CA) (labeled as ACA) using a combination of UV photopolymerization and phase conversion. The other part was a defective ionized UiO66/black phosphorus heterostructure coating (labeled dii-UiO66/BP). The Di-UiO66/BP coating was then combined with the QACA electrolyte to form a bifunctional asymmetric structure. The Di-UiO66/BP coating has a porous structure, which not only provides abundant active sites for the adsorption and catalytic conversion of LiPSs but also enhances the contact area between the electrolyte and the electrode. In addition, the porous structure and high specific surface area of the coating help the electrolyte to form a uniform covering layer on the electrode surface and reduce the voids and defects at the interface. Meanwhile, the low contact angle (9.6°) of the coating indicates good wettability, which enables better contact with the electrode material and further improves the interfacial compatibility. The coating strategy is a key technology for enhancing the compatibility of L-GPE/electrode interface and realizing high-performance solid/semi-solid lithium batteries through physicochemical mechanisms such as modulating wettability, enhancing (electro)chemical stability, inhibiting dendrites, improving mechanical compatibility, and facilitating ion transport. However, its movement from the laboratory to large-scale commercial applications must overcome the technical bottlenecks of the coating process in terms of large-area uniformity, high-speed production, long-term stability, and universality, as well as the economic challenges posed by high-value materials, high-cost equipment, and low production efficiency.

#### In situ Preparation

*In situ* synthesis refers to the method of generating a gel electrolyte directly on the surface of the electrode material or at the electrode electrolyte interface. Unlike the traditional method of first preparing the gel electrolyte and then assembling it with the electrode, in situ synthesis allows the gel electrolyte and the electrode material to be in close contact during the formation process, thus improving the interfacial properties between them. During in situ synthesis, the gel electrolyte is formed directly on the electrode surface or inside the electrode (Fig. 7e). This allows the gel electrolyte to be in close contact with the electrode material, filling pores and defects on the electrode surface and forming a continuous, uniform interfacial layer. This close fit reduces the interfacial resistance and facilitates ion transport between the electrode and the electrolyte. Gao and coworkers prepared GPEs via* in-situ* polymerization and gelation of pentaerythritol tetraacrylate (PETEA) and cellulose acetate propionate (CAP) in a liquid electrolyte (Fig. 7f–h) [127]. The *in-situ* polymerization strategy allows for the construction of stable electrode electrolyte interfaces, which in turn reduces the interfacial reactions. In addition, a hydrogen bond will be formed between PETEA and CAP, which together with the multi-side-chain structure of PETEA can construct a stable cross-linked polymer structure, which provides a channel for Li^+^ migration and promotes the uniform deposition of lithium, facilitating a strong SEI. The robust SEI film has abundant LiF as a barrier, which effectively inhibits Li dendrimer and reduces the decomposition of carbonate solvents, thus ensuring interfacial stability during cycling. As shown in Fig. 7i, the gel shows a lower activation energy (0.040 eV), which indicates a faster lithium-ion migration rate, which in turn indicates a better compatibility of the gel electrolyte. *In-situ* synthesis strategy fundamentally improves the interfacial compatibility of L-GPE with electrodes through physicochemical mechanisms such as elimination of physical gaps, formation of chemically bonded/strongly interacting interfaces, construction of high-quality SEI/CEI, and optimization of ion transport paths. However, its movement from the laboratory to large-scale commercial applications must overcome technical and economic challenges such as high process complexity, difficult control, expensive material costs, poor compatibility with existing production lines, and limited production efficiency. It is the main obstacle to its move to large-scale commercial applications. The technical bottlenecks of the coating process in terms of large-area uniformity, high-speed production, long-term stability, and generalizability, as well as the economic challenges posed by high-value materials, high-cost equipment, and low production efficiency, must be overcome.

### Thermal Stability

Thermal stability is a key property that guarantees the safe operation of L-GPE at high temperatures or under extreme operating conditions. In lithium batteries, insufficient thermal stability will lead to electrolyte decomposition, phase change of anode and cathode materials, or interfacial failure, which in turn will lead to thermal runaway or even explosion. Traditional liquid electrolytes are flammable and volatile, while L-GPE needs to achieve high-temperature resistance and flame retardancy through material design and component optimization.

#### Heat Resistant Additive

**Fig. 8:**
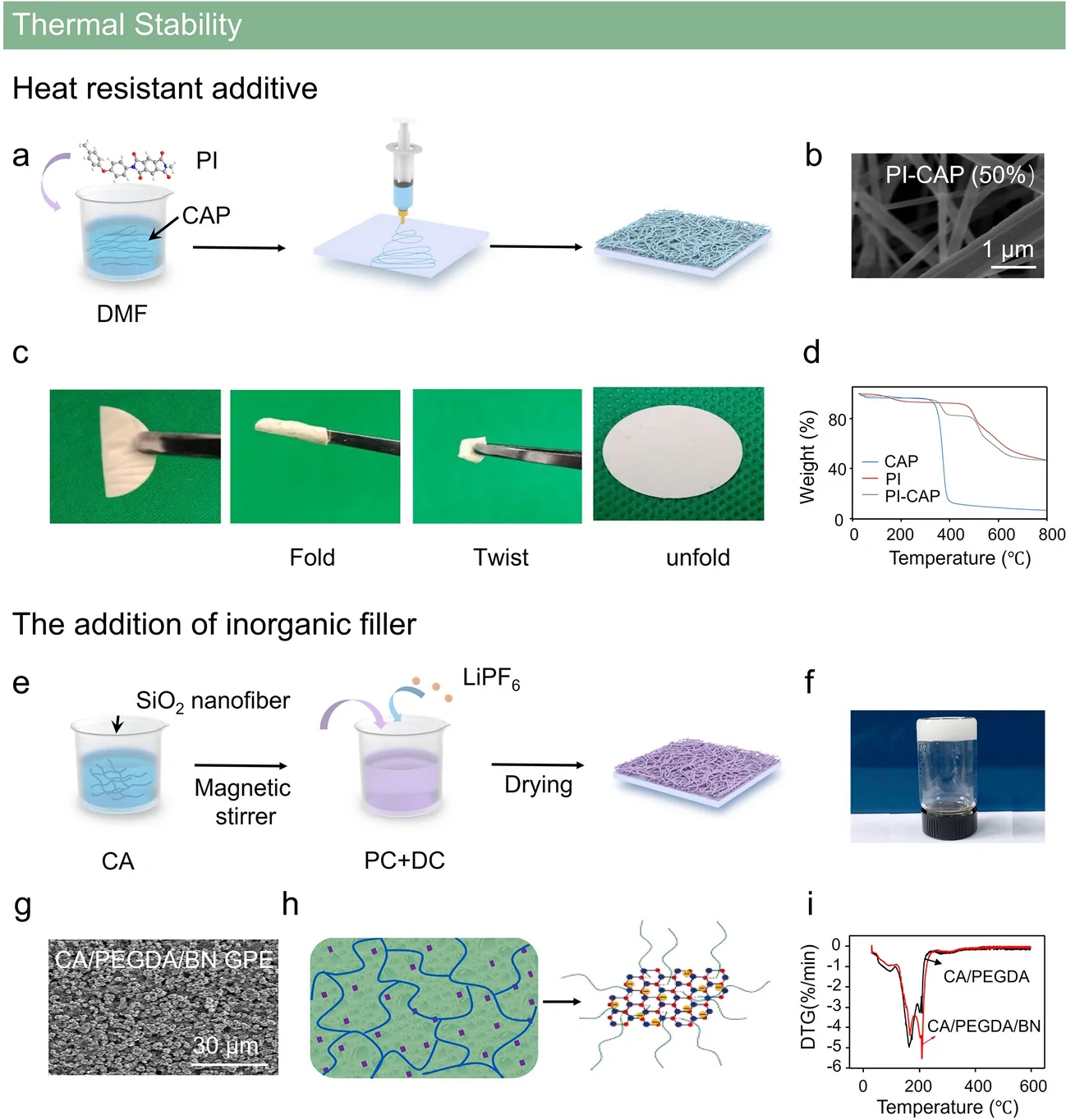
Engineering strategies for improving the thermal stability of L-GPE. **a** Schematic illustration of gel electrolytes through the introduction of a heat-resistant additive. **b** SEM images of PI-CAP. **c** Photo images of PI-CAP membrane via folding, twisting, and unfolding. **d** TG curves of PI, CAP, and PI-CAP membranes. (Reproduced with permission from Ref. [137], Copyright 2023, Springer Nature). **e** Schematic illustrations of gel electrolytes through the addition of inorganic filler. **f** Photo images of PEGDA. **g** SEM image of PEGDA. **h** Schematic diagram of the Lewis acid–base interaction between BN and polymer or anion. **i** DTG curves of GPEs. (Reproduced with permission from Ref. [132], Copyright 2021, Elsevier)

Polyimide (PI) is considered a promising alternative to liquid electrolytes due to its excellent thermal stability, chemical resistance, insulating performances, and self-extinguishing ability. A novel composite GPE matrix consisting of a combination of highly thermally stable PI and environmentally friendly cellulose acetate propionate (CAP) nanofibers was developed by Gao and coworkers [137] (Fig. 8a, b). Polar functional groups on the surface of CAP improve the wettability of the electrolyte and provide good thermal/mechanical properties by hydrogen bonding with rigid PI chains (Fig. 8c). The results show that the prepared PICAP composites have ultra-flexibility, high mechanical strength (7.1 MPa), and excellent thermal stability (over 200 °C) (Fig. 8d). PVDF itself is a semi-crystalline polymer with a regular structure of the crystalline regions and tightly aligned molecular chains, and the disruption of the crystalline regions during heating leads to a drastic change in the material properties. When HFP is doped, the molecular structure of HFP is different from that of PVDF. It will destroy the regular arrangement of the molecular chain of PVDF, making the degree of crystallinity decrease. The increase in amorphous region and the activity space of molecular chain can better absorb and disperse the heat, avoiding the significant decrease in material performance due to local heat concentration, thus improving the thermal stability of gel electrolyte. To prepare gel electrolytes with high thermal stability properties, Wei and coworkers prepared safe GPE with superior mechanical properties and high thermal stability by introducing high-strength lithophilic cellulose acetate (CA) into PVDF-HFP gel electrolyte [131]. Thermogravimetric analysis revealed that the PFP-CA membrane retained 17.71 wt% residual mass at elevated temperatures, significantly higher than the 12.17 wt% residual mass of the pure PFP membrane. It indicates that the combination of CA and PVDF-HFP effectively improves the electrolyte's thermal stability, highlighting the beneficial role of lithophilic cellulose derivatives in enhancing high-temperature resistance. The introduction of heat-resistant additives is an effective strategy to enhance the thermal stability of L-GPE. However, the cost of the additives and the compatibility of the additives with the L-GPE system are still faced in practical applications. Overcoming these challenges requires the development of new additives that are multifunctional, efficient, low side-effect, and low-cost, the optimization of their compounding strategies, the design of smarter and more responsive additives, and the continuous improvement of the process to achieve economic production at scale.

#### Addition of Inorganic Filler

Grafting inorganic nanofillers onto biopolymer chains can provide many benefits (Fig. 8e). On the one hand, it can reduce the possibility of agglomeration and clustering and improve ionic conductivity. On the other hand, incorporating grafted nanostructures can enhance the thermal stability of GPEs, thereby increasing their tolerance to temperature variations and mitigating degradation risks. For example, the grafting of g-SiO_2_ nanofibers disrupts the intermolecular interactions, which influences the stacking configuration of polymer chains. This disruption increases molecular entropy and promotes segmental mobility of the polymer matrix. Das and coworkers grafted silica nanofibers onto cellulose acetate (CA)-based gel electrolytes [138]. The decomposition temperature range in the first stage increased from the initial 264–290.53 to 255–346 °C with the increase of g-SiO_2_ nanofiber concentration; this indicates the improvement of the thermal stability of NGPE. This indicates an improvement in the thermal stability of NGPE. In the second stage, with the incorporation of g-SiO_2_ nanofibers, the temperature range increased to 622 ºC. Compared with pure CA, the thermal stability was significantly better. Layered boron nitride (BN), a typical inorganic filler with Lewis acidic properties, can form intermolecular forces with GPEs to enhance the properties of CA-based GPEs. Liu and coworkers prepared gel polymer electrolytes (GPEs) by combining cellulose acetate (CA) via an in situ thermal polymerization method using layered boron nitride (BN) filler as a reinforcing agent and poly (ethylene glycol) diacrylate (PEGDA) as a cross-linking agent (Fig. 8f, g) [132]. The BN filler was homogeneously dispersed in the crosslinked GPEs with excellent anion trapping ability, which not only interacted with the polymer matrix but also with the liquid electrolytes (Fig. 8h). The BN fillers inhibit the evaporation of organic solvents at low temperatures and accelerate the decomposition of organic solvents at high temperatures. The maximum decomposition rate of organic solvents in CA/PEGDA GPE was 3.3% min^−1^ and that of CA/PEGDA/BN GPE was 5.6% min^−1^ when the temperature reached 210 ºC. The weight loss rates of CA/PEGDA GPE and CA/PEGDA/BN GPE were 72.9% and 67.8%, respectively (Fig. 8i). The results show that the thermal stability of CA/PEGDA/BN GPE is better than that of CA/PEGDA GPE. Compared with the liquid electrolyte, the thermal stability of CA/PEGDA/BN GPE is much better, and it can satisfy the thermal safety requirements of polymer LIBs. The introduction of inorganic nanofillers is a powerful strategy to enhance the thermal stability of L-GPE. However, the biggest bottleneck is the uniform and stable dispersion of nanofillers and the achievement of good filler–polymer interfacial compatibility. Therefore, there is a need to develop low-cost, easily dispersible, functionalized (*e.g.*, with high ionic conductivity) nanofillers.

## Applications of L-GPE in Energy Storage

**Fig. 9:**
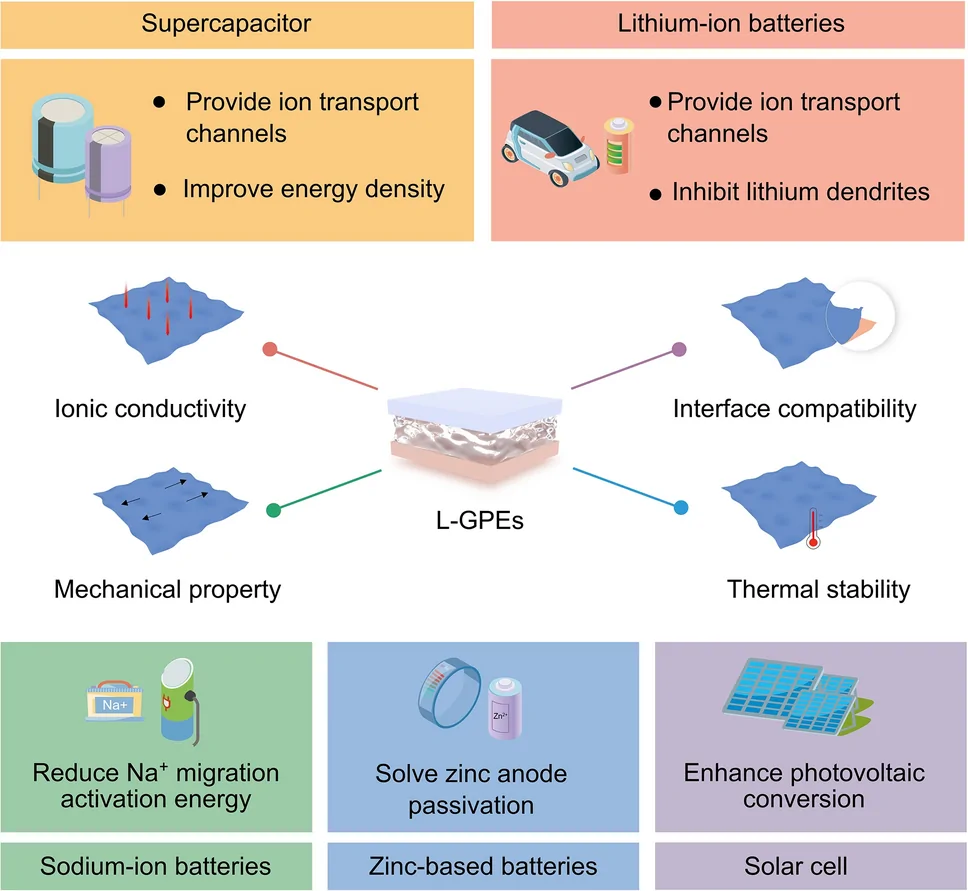
Schematic summary of key requirements and applications of L-GPEs in energy storage applications

Lignocellulose-mediated gel polymer electrolytes (L-GPEs) exhibit distinctive characteristics, such as high ionic conductivity, excellent mechanical flexibility, good interfacial compatibility, and remarkable thermal stability [139]. Precisely due to these appealing properties, substantial research has been conducted on the diverse applications of L-GPEs, and there is a growing interest in their utilization for sustainable energy storage devices. In particular, the development of L-GPEs has significantly broadened the forms, scenarios, and performance in energy storage applications, including supercapacitors, LIBs, SIBs, ZIBs, and solar cell (Fig. 9). Notably, the common features of L-GPEs across these applications are worthy of in-depth discussion. Briefly, the shared characteristics of L-GPEs for these energy-related applications include efficient ionic transport, durable mechanical properties, stable chemical structure, and reliable thermal resistance. Additionally, L-GPEs need to possess specific properties for certain applications. For LIBs and SIBs, L-GPEs are required to have excellent electrochemical stability and compatibility with alkali metal ions to suppress side reactions and electrode degradation. For ZIBs, L-GPEs should have the ability to regulate zinc ion migration pathways, suppress zinc dendrite growth, and maintain structural integrity in mildly aqueous environments. In supercapacitors, L-GPEs need to provide high surface area interfaces and fast ion diffusion kinetics for rapid charge–discharge processes. For solar cells, L-GPEs with tailored optical transparency and ion-conductive networks can facilitate charge separation and transport in hybrid photovoltaic-energy storage systems. In this section, we critically elaborate on the applications of L-GPEs in energy storage through the most recent and representative cases, highlighting their synergistic effects with different electrode materials and device architectures.

### Supercapacitors

Supercapacitors are energy storage devices that combine the superior power density of traditional capacitors with the excellent energy density of batteries, their core advantages lie in fast charge/discharge, long cycle life, and wide temperature region adaptability, and they are widely used in electric vehicles, smart grids, portable electronic devices, and other fields [140, 141]. Supercapacitors typically consist of two electrodes (anode and cathode) separated by an electrolyte (aqueous or organic) and a separator that allows ion transfer while maintaining electrical insulation between the electrodes [142]. Based on the energy storage mechanism, SCs can be classified into two types: electric double-layer capacitors (EDLC) and pseudocapacitance [141]. In double-layer capacitors, the charge is stored mainly through the double layer formed at the electrode/electrolyte interface, which is dominated by physical adsorption and has a fast charging and discharging rate [143]. In pseudocapacitance, the charge is stored mainly through fast reversible redox reactions on or near the electrode surface, and the energy density is higher than that of EDLC. It is widely recognized that the energy and power densities of SCs are proportional to the square of the total cell voltage, which is governed by the electrolyte decomposition voltage [144]. Therefore, the energy density of SCs is greatly influenced by the electrolyte. To achieve superior overall performance, gel polymer electrolytes (GPEs) have attracted substantial research attention in recent years, driven by the rapidly increasing demand for diverse electronic products. Compared with conventional GPEs, L-GPEs have been extensively studied in SCs in recent years due to their natural degradability, biocompatibility, excellent mechanical properties, and low cost. In addition, the application of L-GPEs in the field of flexible electronics has conferred its properties (*e.g*., high toughness, flexible adaptability, and environmental friendliness).

In order to design a gel electrolyte suitable for flexible energy storage devices, the central point is long-lasting mechanical properties. However, current gel electrolytes for supercapacitors have poor mechanical properties due to insufficient cross-linking. In contrast, the structural strategy of double-reticulated hydrogels has been shown to be an effective approach to enhance their mechanical strength. Therefore, from this aspect of research, Chen and coworkers designed an adhesive hydrogel electrolyte using cellulose nanocrystals (CNCs) as a biopolymer nano-reinforcing agent and through the introduction of hydrophobic carbon chains as a remote physical cross-linking agent in the hydrogel matrix [145]. The results showed that CNC-G-12 had the best mechanical properties (210.98 kPa stress resistance and 2204% strain) after 12 h immersion in 1 M potassium hydroxide. In addition, the hydrogel electrolyte showed superior adhesion on the surfaces of both non-conductive and conductive materials including cardboard, leather, carbon film, and carbon cloth. The conductivity of the hydrogel electrolyte was up to 0.207 ± 0.005 S cm^−1^. Notably, the hydrogel electrolyte showed good electrochemical performance when assembled into a coin cell supercapacitor with a single activated carbon sheet as the electrode. The capacitance reached 67.31 F g^−1^ at 0.05 A g^−1^, and the capacitance retention was almost 100% after 2200 cycles at 0.1 A g^−1^.

**Fig. 10:**
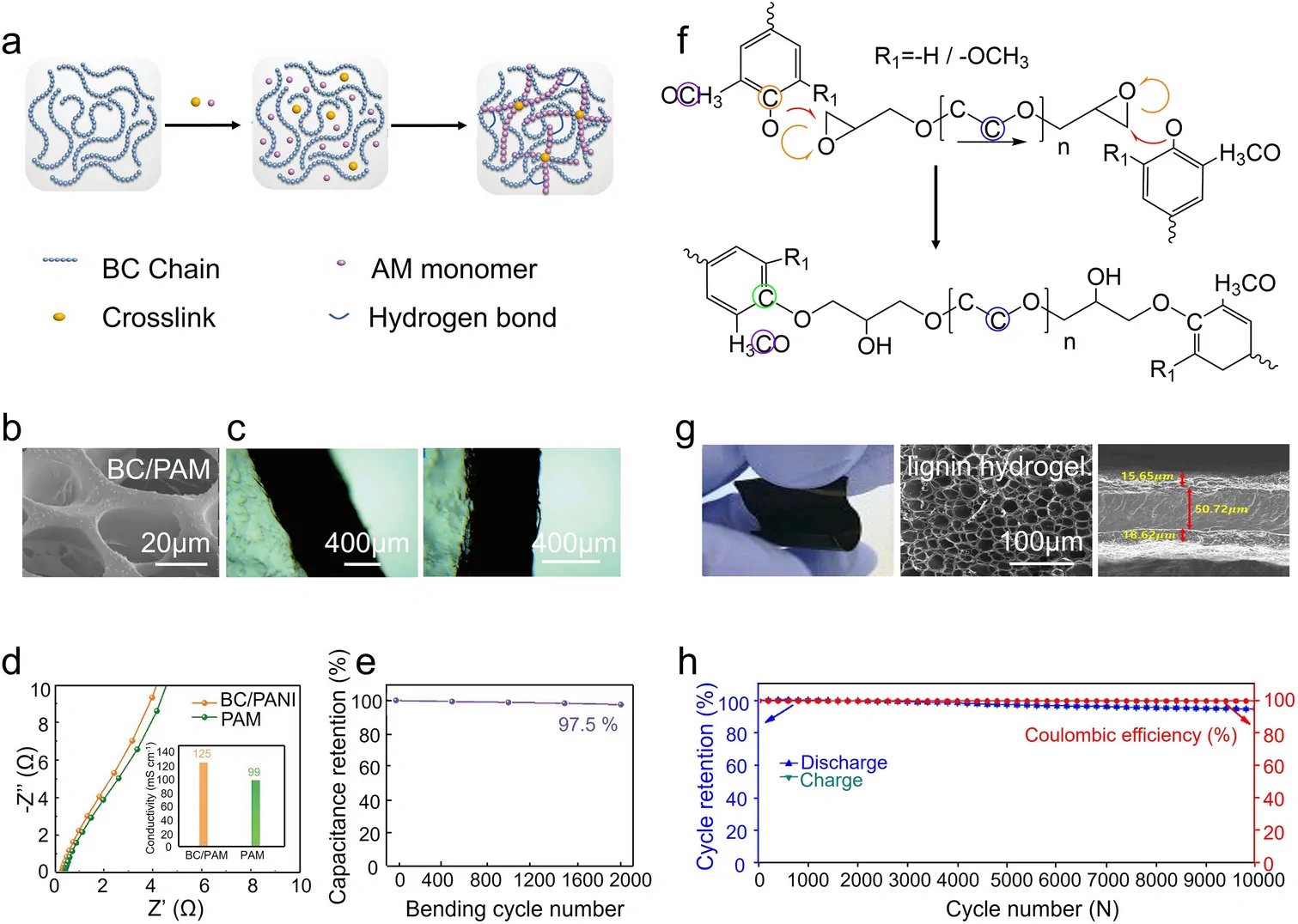
L-GPEs gel electrolyte for supercapacitor. **a** Schematic illustration of the BC/PAM hydrogel. **b** SEM images of the BC/PAM hydrogel. **c** Cross-sectional images of the electrode–electrolyte interface and a small fan driven by four-unit ASCs. **d** Electrochemical impedance spectroscopy plots and of BC/PAM and PAM hydrogel. **e** Cyclic voltammetry curves of all-solid-state symmetric devices; (Reproduced with permission from Ref. [147], Copyright 2021, Wiley–VCH). **f** Cross-linking reaction of the lignin with PEGDGE. **g** Digital photos and SEM images of PEGDGE. **h** Long-term cycle stabilities and Coulombic efficiency of lignin-based SCs at 5 A g^−1^. (Reproduced with permission from Ref. [149], Copyright 2019, The Royal Society of Chemistry)

Bacterial cellulose (BC) has attracted extensive research interest in the field of materials due to its ultrafine interconnected nanofiber network, superior tensile strength, abundant oxygen functional groups, and biocompatibility [146]. The mechanically robust nanofibrous network of BC imparts high mechanical strength to the gel electrolyte without compromising its flexibility, while stabilizing the ion transport channels. Therefore, inspired by the unique properties of BC, Li and coworkers reported a hydrogel electrolyte (BC/PAM) of BC nanofibers composite with polyacrylamide (PAM) [147]. In this structure, a three-dimensional hydrogel matrix was formed between the hydroxyl groups of BC and the amino groups of PAM through physical interactions (Fig. 10a). Notably, the added BC does not alter the original microstructure of PAM but is uniformly dispersed in the PAM matrix (Fig. 10b). The results showed that the presence of BC could significantly improve the mechanical properties of BC/PAM hydrogels. Its tensile strength can reach 330 kPa, which is higher than that of pure PAM (70 kPa). In addition, PANI/RGO/PMFT and BC/PAM have high interfacial compatibility due to hydrophilic interactions and hydrogen bonding (Fig. 10c). This feature gives the gel an excellent ionic conductivity of up to 125 mS cm^−1^ (Fig. 10d). Supercapacitor devices assembled from it have a capacitance retention of 97.5% when bent for 2000 cycles (Fig. 10e). When the device was bent from 0° to 45°, 90°, 135°, and 180°, the change of CV curve was negligible and the capacitance remained almost constant.

However, at low temperatures, the poor conductivity of the gel electrolyte will inevitably fail to meet the requirements of the energy storage device equipment. Therefore, the development of eco-friendly hydrogels integrating antifreeze capability, electrical conductivity, and superior mechanical properties plays a pivotal role in advancing technological progress and facilitating the broad implementation of wearable supercapacitors. Therefore, for high-frequency response requirements, the porous structure of lignocellulosic materials can provide abundant ion channels to further enhance the ionic conductivity; and its flexible network can also reduce the interfacial resistance fluctuations during charging and discharging. From this research direction, Zhang and coworkers synthesized a conductive hydrogel with good freezing and dehydration resistance using cellulose and lithium chloride [148]. The hydrogel was synthesized by polymerization of acrylamide monomer (AM) in the presence of water-soluble cellulose acetate (WSCA) and lithium chloride. The results showed that the hydrogel electrolyte remained soft and pliable at 80 ℃, with certain elasticity and electrical conductivity (23.1 S cm^−1^). In addition, supercapacitors assembled with PAM/LiCl/WSCA hydrogel as electrolytes maintained excellent capacitance stability after 500 folding cycles and 10,000 charge/discharge tests. Notably, the assembled capacitors exhibited excellent flexibility and stable electrochemical performance at low temperatures, maintaining 64.64% capacity even at 40 ºC. This simple method of preparing freeze-resistant conductive hydrogel electrolytes provides new ideas and ways to use hydrogels as electrolytes in extremely cold environments.

In recent years, lignin and its derivatives have been investigated for the preparation of gel electrolytes that can be used in flexible energy storage devices [98]. However, their performance has not been able to fulfill both electrochemical and mechanical properties. Therefore, Park and coworkers reported an all-lignin-based flexible supercapacitor by combining lignin hydrogel electrolytes with lignin/polyacrylonitrile nanofiber electrodes (Fig. 10f) [149]. After swelling, the lignin gels transformed the surface dense structure into a porous structure, and the combination of this structure with the chemical cross-linking structure made the gels structurally stable for storing a large number of proton carriers, which further improved the ionic conductivity of the gels (Fig. 10g). The results showed that the assembled supercapacitors maintained high capacitance at different bending angles and exhibited excellent cycling stability of more than 10,000 cycles at 5 A g^−1^ with a Coulombic efficiency higher than 99% (Fig. 10h). Based on the availability of these high-performance flexible SCs, our strategy using renewable biomass-based materials is designed to contribute to the development of sustainable materials for energy storage.

### Lithium-Ion Batteries

As an efficient energy storage device, lithium batteries have become a popular choice for major portable electrical devices, electric vehicles, and wearable electronics in our daily lives due to their high energy density, low charge loss, long cycle life, and lightweight [150]. Generally speaking, Li-ion batteries are mainly composed of a positive electrode, a negative electrode, a diaphragm, and an electrolyte [151]. Notably, most commercial lithium batteries are based on liquid electrolytes. Conventional liquid electrolytes usually consist of organic solvents, lithium salts, and additives. Although liquid electrolytes have high electrical conductivity, there are some unavoidable problems: side reaction occurrence, dendrite growth, design and assembly difficulties, etc. To address the insufficient high-voltage stability and lithium dendrite problem, the aromatic ring structure of lignin enhances the antioxidant property and broadens the electrochemical window against oxidative decomposition at high voltage; while the rigid network constructed by cellulose nanofibers physically blocks lithium dendrite puncture, and at the same time, the branched chain of hemicellulose enhances through the “weak coordination–fast dissociation” mechanism the Li⁺ mobility number, optimizing the ion transport efficiency. In recent years, many researchers have worked on developing gel electrolytes to replace liquid electrolytes. L-GPEs are becoming more and more attractive in the fabrication of flexible batteries due to their non-flammable and non-toxic properties.

Cellulose, as the most widely spread and abundant natural biomass material, has attracted wide interest in the field of polymer matrix materials due to its multiple advantages, such as high density of polar hydroxyl groups, biodegradability, renewability, thermal stability, environmental friendliness, and economic low cost [152, 153]. In recent years, it has been successfully used as a gel polymer for lithium-ion batteries. However, these efforts still suffer from problems such as low ionic conductivity or poor lithium-ion transferability. From this research direction, Du and coworkers prepared a cellulose gel membrane with good mechanical properties and environmentally friendly by solution casting and cross-linking methods [154]. The results showed that the GPE prepared from cellulose membrane with 5% cross-linking agent added had excellent electrochemical properties with an ionic conductivity as high as 6.34 × 10^–3^ S cm^−1^. In addition, the assembled battery had a discharge capacity of 145 mAh g^−1^ after the first cycle and capacity retention of 90% after 50 cycles.

**Fig. 11:**
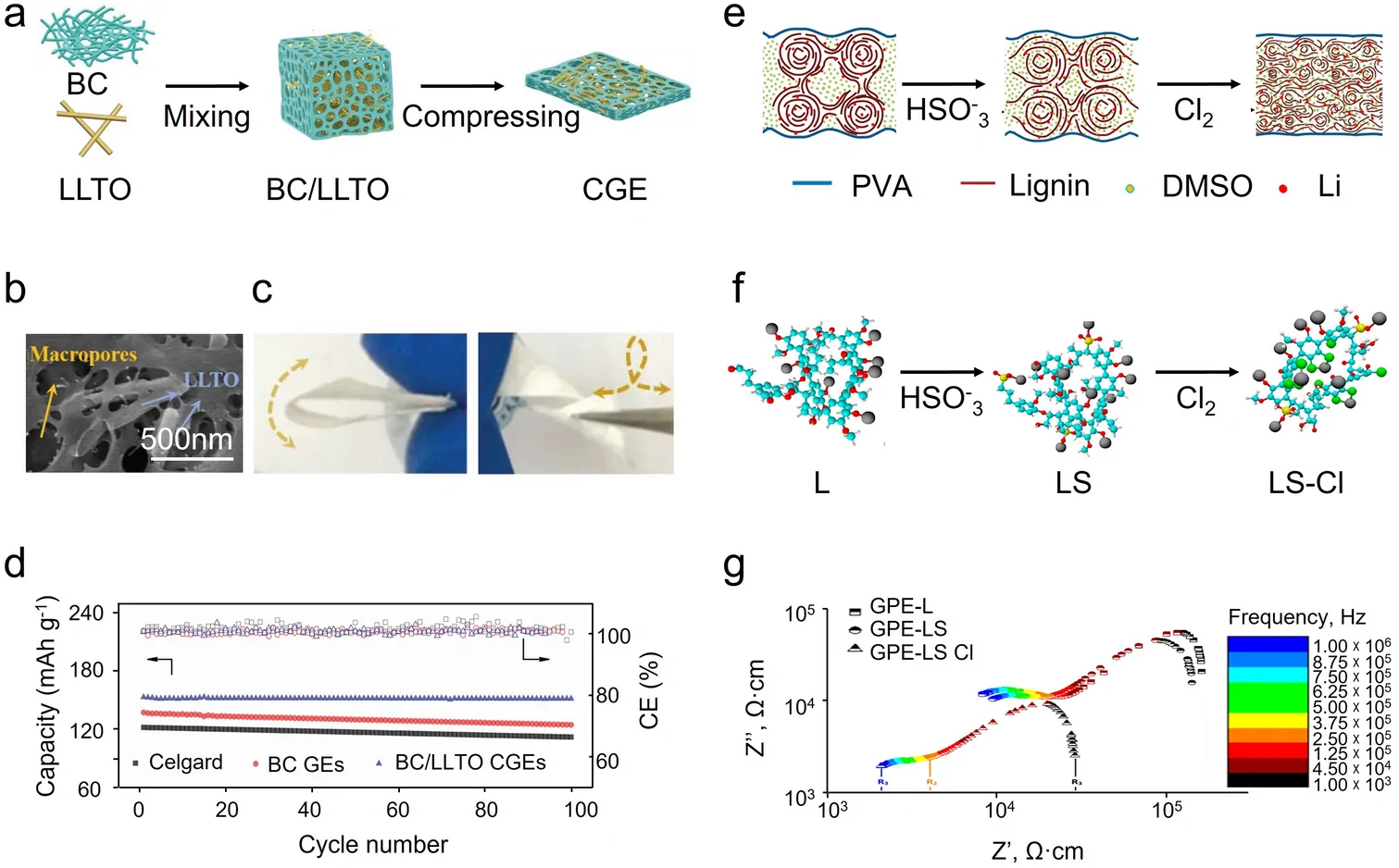
L-GPEs gel electrolyte for lithium-ion batteries. **a** Schematic illustration of BC/LLTO CGEs. **b** SEM image of BC/LLTO CGEs. **c** Photographs showing high flexibility of the BC/LLTO CGE matrix. **d** Cycle performance of BC/LLTO CGE (Reproduced with permission from Ref. [157], Copyright 2019, Wiley–VCH). **e** Schematic diagrams of the preparation of GPE-LS. **f** Fragments of L, LS, and LS-Cl molecules. **g** Fragments of L, LS, and LS-Cl molecules. (Reproduced with permission from Ref. [160], Copyright 2021, MDPI)

Nanocellulose-based GPEs have attracted much attention due to their abundant resources, biodegradability, renewability, and good mechanical strength [155]. However, the high amount of hydroxyl groups in the precursor membranes of GPEs leads to a dense morphology, which severely affects their electrolyte uptake ratio and further electrochemical performances. Therefore, there have been studies on its surface modification by acetylation of pretreated cellulose nanofibrils (CNFs) [156]. This strategy promotes the dissociation of lithium salts by introducing ester groups, and the strong molecular interactions between the ester groups and Li^+^ ions further improve their electrolyte uptake capacity, and the number of hydroxyl groups is reduced, leading to stable battery cycling performance. Notably, the initial discharge capacity of the assembled Li/GPE/LiFePO_4_ battery was 153 mAh g^−1^ after 100 cycles at 0.2 C, with a capacity retention of 88.8%. However, the modulus of these GPEs was still at the megapascal level and the lithium-ion transfer number was limited to below 0.8. However, the modulus of cellulose-based GE is still at the megabase level and the lithium-ion transfer number is limited to below 0.8. Unlike other types of cellulose, bacterial cellulose has higher molecular weight, purity, and crystallinity. Therefore, Ding and coworkers composited bacterial cellulose (BC) and Li_0.33_La_0.557_TiO_3_ nanowires (LLTO NWs) to make an aerogel and prepared gel electrolyte matrices with a highly porous structure with three-dimensional interconnections (Fig. 11a) [157]. The BC host acted as a protective layer, which effectively prevented direct contact between the LLTO NWs and the Li metal, thus eliminating the side reactions between them. Compared with the original cell wall, this cell wall has more macropores, which facilitates the uptake of electrolytes by the CGE matrix (Fig. 11b). The excellent mechanical properties are critical for with-standing the huge compression from cell assembly and blocking Li dendrites. The photographs as shown in Fig. 11c indicate that the BC/LLTO CGE has good flexibility and can withstand various mechanical deformations without rupture. Moreover, the composite aerogel has a Young's modulus of up to 1.15 GPa and a lithium-ion transfer number of up to 0.88. It is well known that inhomogeneous lithium deposition is the root cause of lithium dendrite growth. In the LMB of BC/LLTO CGEs, the synergistic effect of the BC backbone and the LLTO NWs allowed the Li dendrite growth to be effectively suppressed and blocked. The assembled half-cells showed the highest discharge capacity at all current densities and exhibited stable cycling performance with no obvious capacity degradation for 100 cycles at 0.2 C (Fig. 11d).

The development of lignin (L), the second most abundant biopolymer on earth, will reduce the environmental impact and provide an energy storage and conversion device for cheap and readily available primary resources [158, 159]. However, lignin mainly contains weak polar groups such as phenolic hydroxyl (Ar-OH) and methoxy (O–CH₃), which are unable to effectively dissociate lithium salts (*e.g*., Li⁺ with the corresponding anion), resulting in a low free lithium-ion concentration. Through chemical modification, lignin can also be imparted with ionic conductivity. From this research direction, Shabanov and coworkers sulfonated and chlorinated lignin, and then prepared gel electrolytes by compounding the modified lignin with polyvinyl alcohol (PVA, 20 wt%) (Fig. 11e). After sulfonation, sulfonic acid groups (-SO₃^−^) were introduced, and Li⁺ was combined with sulfonic oxygen and phenolic hydroxyl groups to shorten the bond length and increase the polarity. Further after chlorination, part of the benzene ring is replaced by chlorine atoms and ligand bonds are formed with Li⁺. The introduction of chlorine significantly increases the molecular dipole moment and the polarization effect is more pronounced (Fig. 11f). Therefore, sulfonation and chlorination significantly enhance the lignin molecular polarity and promote lithium-ion dissociation and migration. Notably, the homogeneous network of the modified electrolyte reduced the interfacial and bulk impedance (Fig. 11g). The conductivity can reach 2.48 × 10^–4^ S cm^−1^, which is comparable to most commercial polymer electrolytes. This study opens up a new avenue for the application of lignin in energy storage, and the developed GPE-LS-Cl can significantly improve the charge/discharge rate, current density, and cycling stability of lithium-ion batteries while meeting the demands of environmental protection and low cost.

### Sodium-Ion Batteries

Compared to lithium, sodium has the advantages of abundant reserves, ease of mining, and large-scale production. Given the abundance of sodium resources, sodium-ion batteries are receiving increasing interest in large-scale energy storage, and they may be suitable as one of the alternatives to lithium-ion batteries [161]. However, since the radius of Na^+^ is larger than that of Li^+^, the diffusion of Na^+^ is slower than that of Li^+^ during charging and discharging, resulting in low diffusion coefficients, large volume expansion, and poor cycling performance. To address the problem of high Na⁺ migration resistance, the vertical porous channels formed by oriented freezing of lignocellulose can shorten the migration path, and its hydroxyl group and Na⁺ moderate coordination energy can reduce the migration energy barrier, so that the ionic conductivity can be improved; hemicellulose's flexible chain segments can also alleviate the space of the solvated shell layer of Na⁺ to further promote conduction. Site resistance, further facilitating the conduction. Thus, it is necessary to design reasonable materials to allow fast diffusion kinetics. In addition, sodium-ion batteries suffer from a number of shortcomings, including flammability, electrolyte leakage, and dendrite growth. In this respect, GPE is considered to be a reliable alternative to conventional organic liquid electrolytes, with special advantages in inhibiting the formation of sodium dendrites. Lignocellulose derived from natural biomass can improve salt solubility due to its hydrophilic functional groups (*e.g.*, hydroxyl and carboxyl groups) that have a good affinity for polar solvent molecules [162]. Therefore, LGPEs with non-flammable and non-toxic properties are becoming increasingly attractive for the fabrication of flexible batteries.

**Fig. 12:**
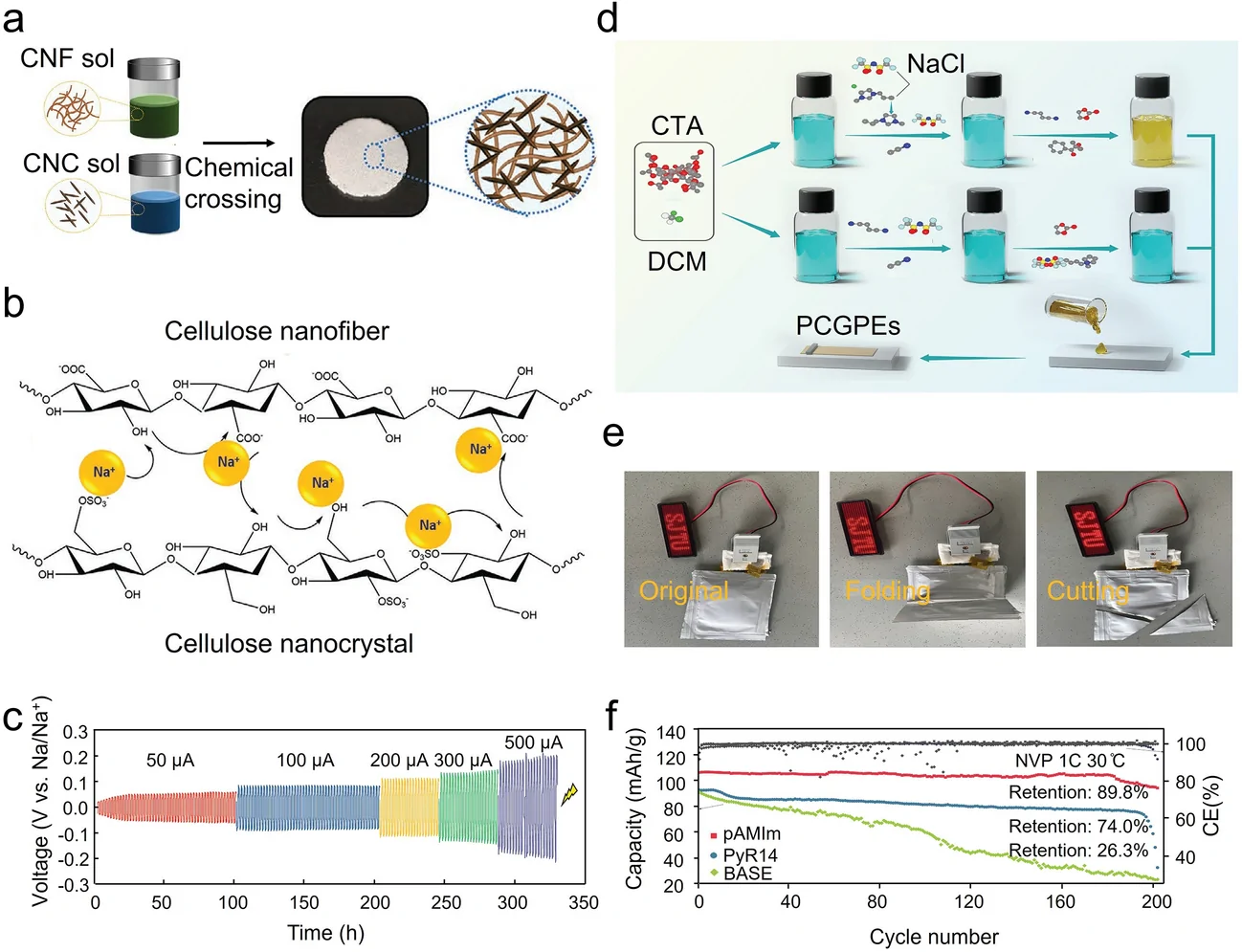
L-GPEs gel electrolyte for sodium-ion batteries. **a** Schematic illustration of the preparation of CNC/CNF cryogel. **b** Schematic illustration of the Na.^+^ conduction mechanism across the nanocellulose GPE. **c** Room temperature voltage versus time curves for a symmetric Na/Na cell; (Reproduced with permission from Ref. [165], Copyright 2022, Wiley–VCH). **d** Preparation route of PCGPEs. **e** Illustration of NVP-Na pouch cells lighting a light board. **f** Cycling performance of NVP/Na cells. (Reproduced with permission from Ref. [166], Copyright 2024, Wiley–VCH)

Cellulose nanocrystals, as a kind of 100–1000 nm long needle-like nanoparticles (2–20 nm in diameter), can form mesoporous structures with hierarchical arrangement (pore sizes from 2 to 50 nm) [163]. This mesoporous structure achieves uniform ion transfer between electrodes by enhancing ion migration, which in turn leads to a stable and reversible ion plating and stripping process, effectively inhibiting dendrite formation. However, its mechanical properties are deficient, and the mechanical stability of CNC-based materials can be improved by blending with other cellulosic materials to enhance the electrochemical performance [164]. Considering these aspects, Mittal and coworkers used biodegradable cellulose nanocrystals and cellulose nanofibrils to prepare gel polymer electrolytes [165] (Fig. 12a). The results showed that due to the synergistic effect of CNCs and CNFs, the ionic conductivity of this electrolyte could reach 2.32 mS cm^−1^ with a Na^+^ transfer number of 0.637 (Fig. 12b). The optimal ratio of cellulose nanocrystals to cellulose nanofibers led to the formation of a mesoporous layered structure, based on which a sodium-ion battery assembled at 1C current density exhibited an energy density of 240 Wh kg^−1^ and a specific capacity of 69.7 mAh g^−1^ after 50 cycles at 1 C current density (Fig. 12c). This gel-polymer electrolyte system is expected to open-up new avenues for sustainable energy storage technologies beyond lithium-ion batteries. Cellulose triacetate (CTA) is a readily available cellulose derivative known for its strong mechanical properties and chemical stability as well as its high dielectric constant that promotes ion dissociation. In this regard, Zhao and coworkers developed a polymer-ionic gel polymer electrolyte based on CTA (Fig. 12d) [166]. In the structure of this electrolyte, CTA serves as the main backbone with excellent mechanical properties. Several oxygen groups are present in the CTA, which positively interact with sodium ions, and thus the sodium ions can be transported and dissociated more efficiently, which is essential for the efficient operation of the battery. The reliability and safety of the assembled pouch battery unit can be seen in the fact that the batteries continue to discharge even after folding and cutting operations (Fig. 12e). The assembled battery maintained 89.8% capacity retention of pAMIm by the 200th cycle. The capacity was 106.0 mAh g^−1^ at 0.1 °C and 95.1 mAh g^−1^ at 2 °C, which is 89.7% capacity retention (Fig. 12f). These results emphasize the potential of this novel electrolyte in advancing sodium-metal battery technology. Like plant cellulose, BC is biocompatible, degradable, and non-toxic. The glucose monomer and linear chain structure of BC facilitate salt decomposition and electrolyte solvent attachment. Liu and coworkers developed a high-strength gel polymer electrolyte (BC-GPE) based on bacterial cellulose network [167]. The electrolyte has a rich interwoven structure of three-dimensional ion transport channels, which gives it high strength (tensile strength of 36 MPa and maximum strain of 31.23%). In addition, the self-assembled sodium-ion battery has a high multiplicity performance of 1250 cycles and a long cycle life with a capacity decay rate of only 0.005%.

### Zinc-Based Batteries

In recent years, zinc-ion batteries have received widespread attention. Zinc metal is abundant, has low toxicity, is easy to handle, and has higher energy among the metallic elements that can be stabilized in aqueous solution [168]. Compared with other types of batteries, zinc-ion batteries have superior electrochemical performance. In general, it is mainly reflected in the following advantages: (1) higher energy density and power density; (2) good multiplier performance; (3) low-cost and simple manufacturing process; and (4) environmental protection and safety [169, 170]. Therefore, zinc-ion batteries are expected to become an efficient energy storage device for the next generation of portable electronic devices [171]. However, it has the problems of leakage of water-electrolyte and instability of zinc anode, which leads to few practical applications [172]. To address these challenges, lignocellulosic materials are modified by acetylation to reduce hydrophilicity, stabilize the interfacial pH at acidic, and inhibit HER; their nanopores uniformly distribute Zn^2^⁺ fluxes through sieving effects, which combined with the weak adsorption of hydroxyls guides the homogeneous deposition, resulting in a longer zinc-symmetric battery cycle life [173]. In recent years, lignocellulose-based gel electrolytes have been widely used as an emerging environmentally friendly material for the fabrication of durable and multifunctional zinc-ion batteries.

**Fig. 13:**
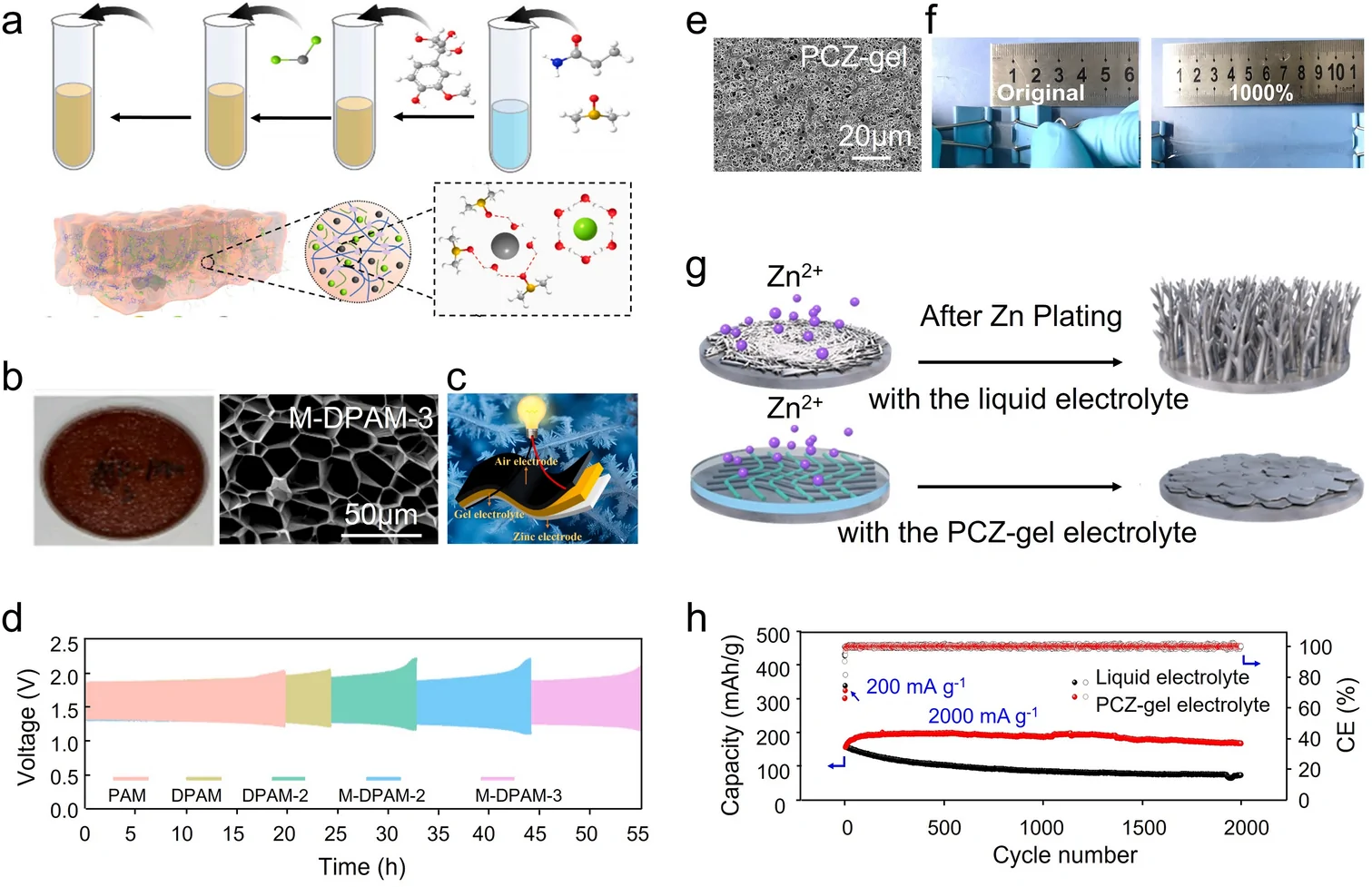
L-GPEs gel electrolyte for zinc-based batteries. **a** Schematic image of preparation of M-DPAM-3 hydrogel electrolyte. **b** Image and SEM image of M-DPAM-3 hydrogel electrolyte. **c** Structure diagram of flexible Zn-air battery. **d** Cycling performances of the flexible batteries; (Reproduced with permission from Ref. [179], Copyright 2025, Elsevier). **e** SEM image of PCZ-gel. **f** Photographs of stretching of PCZ-gel. **g** Schematic diagrams of the Zn plating behaviors. **h** Long-term cycling performance at 2000 mA g^−1^ (Reproduced with permission from Ref. [178], Copyright 2023, Wiley–VCH)

Lignin has a certain electron-donating ability, enabling it to chelate with Zn^2+^ ions and displace water molecules in their coordination sphere, thereby suppressing the formation of [Zn(H₂O)₆]^2+^ solvated complexes. Through this modification of the zinc anode surface solvation structure, the growth of zinc dendrites is effectively mitigated, concurrently enhancing the cycling stability of the zinc electrode. Therefore, Zhong and coworkers prepared an M-DPAM-3 hydrogel electrolyte by using a high concentration of zinc chloride as a pore-forming agent and salt additive, combined with macromolecule lignin as a restorative agent (Fig. 13a). M-DPAM-3 hydrogel was synthesized through the introduction of the cross-linking of lignin with PAM; the disordered network structure was altered to have a rich porous structure while maintaining an orderly arrangement with good electrochemical and mechanical properties (Fig. 13b). Figure 13c shows the schematic structure of a zinc-air battery, which consists of a zinc electrode, a gel electrolyte, and sequentially stacked air electrodes. M-DPAM-3 exhibits an excellent performance with an ionic conductivity of up to 440.91 mS cm^−1^. This significant conductivity enhancement stems from both its unique molecular backbone structure and the optimized ion transport pathways within the electrolyte matrix. Notably, compared to conventional PAM hydrogels, the cycling lifespan of M-DPAM-3 hydrogels is extended by 175% over 55 h of continuous operation (Fig. 13d). The improvement is ascribed to the synergistic action of zinc chloride and lignin on the hydrogel network: zinc chloride increases pore formation, while lignin reinforces structural stability, collectively boosting ionic conductivity and water retention capacity.

Highly amorphous cellulose is environmentally friendly, biodegradable, and processable, and can be used as a robust and durable solid electrolyte for aqueous structured batteries [174]. Lim and coworkers reported a novel non-fibrous, highly amorphous cellulose polymer electrolyte “Cellyte” [175]. There is a fully coordinated Zn^2+^ hydration shell in cell-4.5, and the cellulose chains on the second shell away from the Zn^2+^ ions can anchor water molecules in the first shell around the Zn^2+^ ions (solvated sheath). As a result, the electrochemical stability and cycling performance of LMOCF//cell-4.5//Zn remain high. The cycle life of the assembled Zn//cell-4.5//LMO-CF full cell exceeded 1000 h and a good capacity retention of 90.6% was obtained. Stable ion transport and power output were observed even under deformation stress and recovery conditions.

Currently, most aqueous zinc-ion batteries (AZBs) cathode materials are mainly proton-participating or even proton-dominated and thus can be prone to interfacial ZSH deposition. The introduction of small acid molecules has become a logical approach to provide a high proton environment. Among them, polymeric acids have high stability and allow slow proton ionization/doping during intercalation/extraction. This property may contribute to a continuous and reversible supply of protons, which can effectively mitigate interfacial problems. From this aspect, Zhang and coworkers prepared carboxylic acid-functionalized hydrogel electrolytes by grafting poly (acrylic acid) (PAA) onto cellulose matrix [176]. The unfavorable deposition of by-products can be effectively eliminated by surface modification. Zinc-ion batteries prepared using this gel electrolyte still provided a specific capacity of 244 mAh g^−1^ after 1500 cycles, with a retention rate of up to 80% relative to its maximum value of 306 mAh g^−1^. Despite the great progress, there are still potential problems such as the crystal orientation of the deposited Zn, which is closely related to the growth of dendrites. Lin and coworkers reported a novel polyacrylamide-poly (ethylene glycol)-diacrylate-carboxymethylcellulose (PMC) hydrogel electrolyte [177]. A large number of amide groups in the PMC hydrogel can guide the deposition of Zn^2+^ in a highly selective orientation. These positive effects result in uniform and dense deposition of the zinc anode during cycling. The battery assembled with it was cycled at a current density of 1.0 A g^−1^ with an initial specific capacity of 380.74 mAh g^−1^ and a capacity retention of 71.1% after 500 cycles. At current densities of 0.1–2.0 A g^−1^, the battery cell has good multiplication performance.

In recent years, cellulosic materials have been used as physical frameworks for hydrogel electrolytes for aqueous zinc-ion batteries, but their electrochemical properties and mechanical strength are still unsatisfactory. A rigid-soft energy-depleting dual network (DN) strategy can enhance the mechanical properties of hydrogels by introducing sacrificial bonds. Notably, in poly (amphiphilic) polymer electrolytes, electrostatic interactions between amphiphilic groups and counterions can establish separated ion transport channels, which can significantly improve the electrochemical performance. Therefore, from this research direction, Zhang and coworkers reported an amphiphilic DN hydrogel electrolyte (PCZ-gel) consisting of PAM, carboxyethyl quaternized cellulose (CEQC), and ZnSO_4_ salts [178]. The counterions of the PCZ-gel with amphiphilic groups from the CEQC chains promote network connections and form a hierarchical and porous structure (Fig. 13e). Due to its unique DN structure, PCZ-gel has high strength and toughness. It can be stretched up to 10 times (Fig. 13f). The amphiphilic groups can homogenize the flux of Zn^2+^ and direct the diffusion of Zn^2+^ ions in a three-dimensional direction (Fig. 13g). As a result, PCZ-gel can effectively inhibit the formation of Zn dendrites, thus realizing an ultra-stable Zn anode. The de Zn//NVO cell constructed with this electrolyte achieves a high reversible specific capacity of 226 mAh g^−1^ at a current of 1000 mA g^−1^ and remains stable after 150 cycles with a capacity retention rate of 84% (Fig. 13h).

### Solar Cell

As a semiconductor device capable of directly converting solar energy to electrical energy, the core function of a solar cell is to directly convert light energy into electrical energy through the photoelectric effect [180]. Its core mechanism relies on the photovoltaic effect-photons excite electron hole pairs in the semiconductor material, which achieves charge separation and generates an electric current through a built-in electric field. Currently, mainstream solar cells include silicon-based cells (monocrystalline silicon, polycrystalline silicon), chalcogenide cells (PSCs), and dye-sensitized solar cells (DSSCs), of which DSSCs and emerging flexible/wearable solar devices have attracted much attention due to their low cost, flexibility, colorful appearance, and high dependence on electrolytes. DSSCs primarily consist of four key components: the photoanode, dye sensitizer, electrolyte, and counter electrode. Although conventional liquid electrolytes have high ionic conductivity (> 10 mS/cm), the problems of easy leakage, volatilization, and unstable interface with electrode materials limit their practical applications. Therefore, the development of solid/gel electrolytes with high ionic transport capacity, mechanical flexibility, and environmental stability has become a research focus in this field. The three-dimensional network structure of lignocellulose can form a rigid skeleton to fix the liquid electrolyte in the gel matrix to avoid leakage [181]. Meanwhile, the intermolecular hydrogen bonding within the lignocellulose can enhance the mechanical properties of the system, which is suitable for the bending and encapsulation needs of flexible DSSCs.

**Fig. 14:**
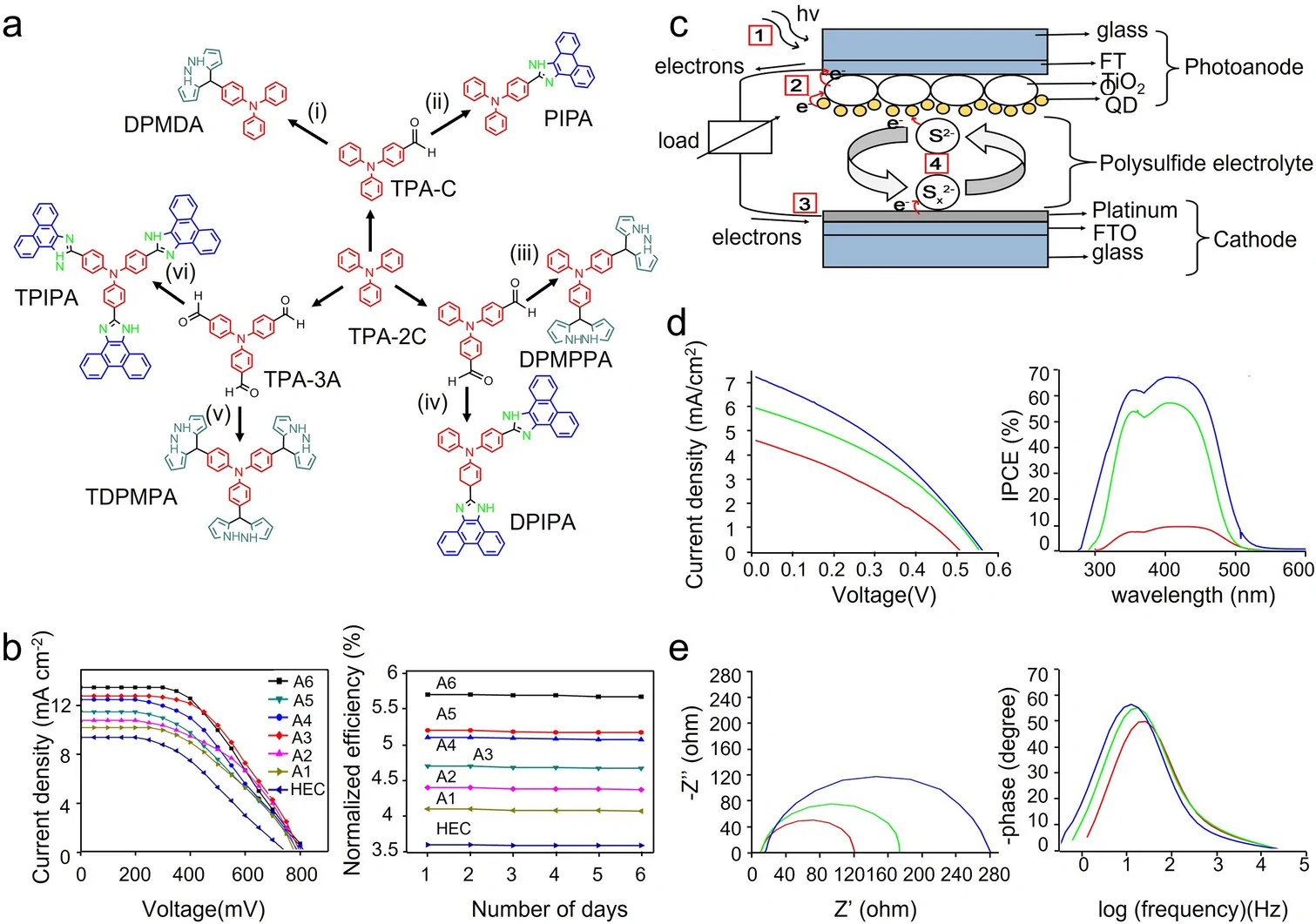
L-GPEs gel electrolyte for solar cell. **a** Schematic image of the dye-sensitized solar cells. **b** I − V curves and stability curves of the gel electrolytes; (Reproduced with permission from Ref. [182], Copyright 2021, ACS). **c** Schematic diagram of QDSSC works. **d** IPCE curves and J-V characteristics of the QDSSC. **e** Nyquist plot and Bode phase plot of the QDSSC. (Reproduced with permission from Ref. [183], Copyright 2016, Springer)

Lignocellulose-mediated gel polymer electrolytes (L-GPEs) provide innovative solutions for solar cell electrolytes by virtue of their 3D porous network structure, tunable functional groups (*e.g*., hydroxyl, carboxyl), and biodegradability. Raja and coworkers combined hydroxyethylcellulose containing I-/I3-redox pair polymer in combination with a triphenylamine core to construct gel electrolyte-based DSSCs (Fig. 14a) [182]. Due to the Lewis alkalinity of the semiconductor, the organic additive of the triphenylamine core was adsorbed on the TiO_2_ surface, thus improving the performance of the DSSCs. In addition, the electrolyte was stable over six days with no significant change in its performance (Fig. 14b). Methyl cellulose is biocompatible and water-soluble and can be chosen as a host polymer for the fabrication of GPEs. Mingsukang and coworkers prepared a methyl cellulose-polysulfide gel polymer electrolyte with an FTO/TiO_2_/CdS/ZnS/SiO_2_/electrolyte/Pt (cathode) structure for use in quantum dot sensitized solar cells (QDSSCs) [183] (Fig. 14c). In this study, the surface was first treated with ZnS and then passivated with SiO_2_. It is shown that the introduction of a passivation layer can significantly enhance the performance of QDSSCs (Fig. 14d). When the ZnS passivation layer structure [TiO_2_/CdS_5_/ZnS_2_/electrolyte/Pt cathode] is employed, the IPCE value of the device is enhanced to 57.24%, and this efficiency index is further increased to 67.20% after further introduction of the SiO₂ passivation layer [TiO_2_/CdS_5_/ZnS_2_/SiO_2_(24 h)/electrolyte/Pt cathode] (Fig. 14e).

## Future and Challenges

**Fig. 15:**
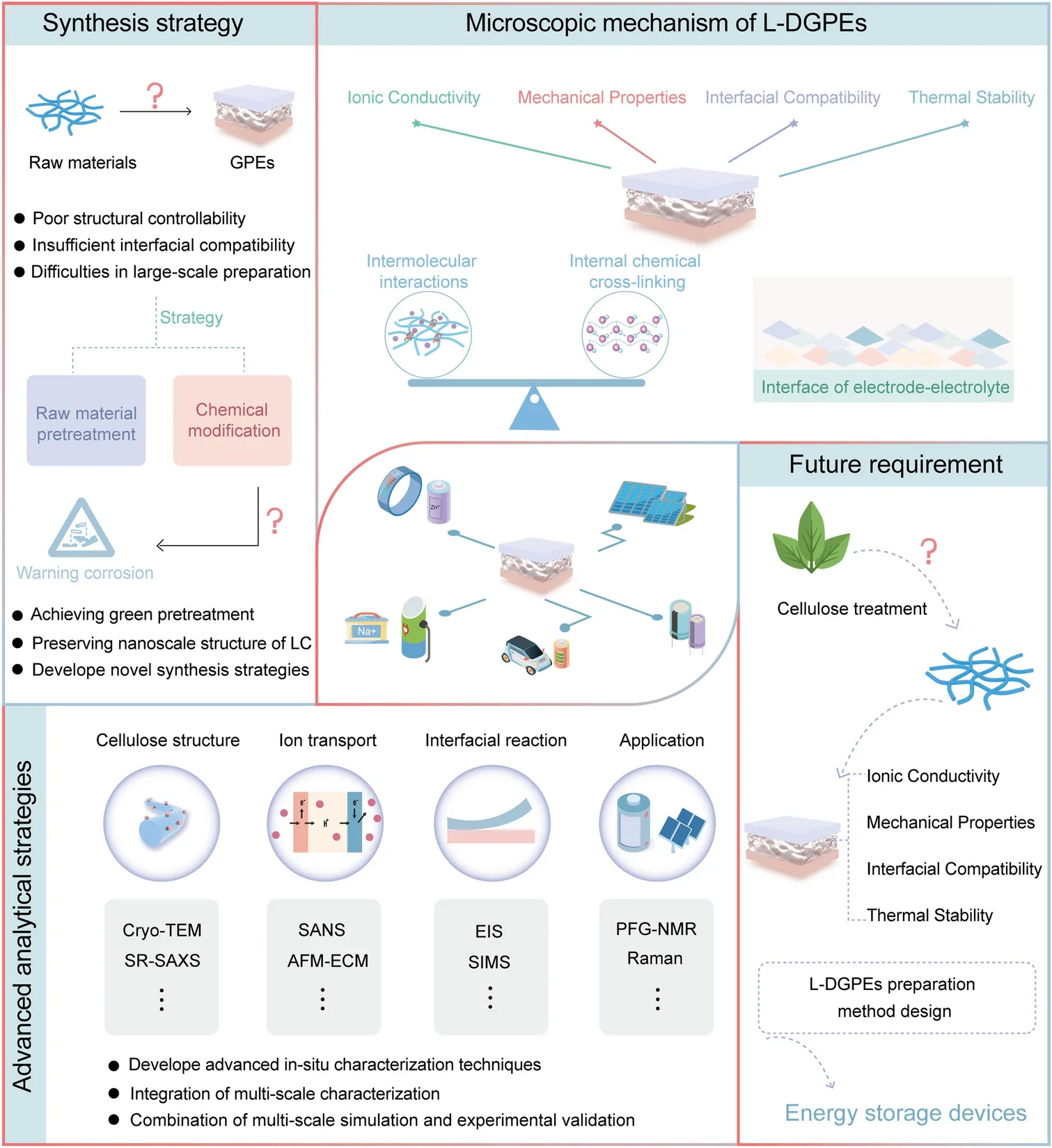
Schematic diagram of the preparation strategies, microscopic mechanism, advanced characterizations, and future development of L-GPE for ESDs

In recent years, lignocellulose-mediated gel polymer electrolytes (L-GPEs) have gained increasing attention as flexible electrolytes for high-performance energy storage devices (ESDs) due to their low cost, biodegradability, environmental friendliness, and excellent mechanical properties. This paper reviews the recent advances in L-GPEs for ESDs, highlighting (i) the major challenges currently facing L-GPEs, (ii) the great potential of lignocellulosic materials as gel electrolytes, (iii) the key elements of L-GPEs for energy storage devices and the effective strategies provided to improve the performance of lignocellulose-based gel electrolytes, and (iv) the potential of L-GPEs in supercapacitors, lithium-ion batteries, and (v) the potential of lignocellulosic materials as flexible electrolytes for energy storage devices (*e.g.*, capacitors, lithium-ion battery cells, sodium-ion batteries, zinc-ion batteries, and solar cells). In this process, the future development of gel electrolytes based on lignocellulosic materials still faces many challenges, and the difficulties and performance enhancement of special L-GPEs still need a lot of effort, so further research directions of L-GPEs to advance the advanced ESDs are as follows (Fig. 15):

(i) ***Awareness of L-GPEs synthesis processes.*** The synthesis process of lignocellulose-based gel electrolytes is usually complex and involves multiple steps, such as raw material pretreatment, chemical modification, and gel formation, resulting in lower production efficiency and higher cost. Therefore, the development of a low-cost, scalable, and environmentally friendly production process is urgently needed for the commercial application of lignocellulose-based gel electrolytes. As the most abundant renewable biomass resource on earth, lignocellulose, with its nanostructures (*e.g*., cellulose nanocrystals, nanofibers), exhibits unique advantages in the field of gel polymer electrolytes (GPEs) due to its high specific surface area, porousness, and degradability. However, there is a significant contradiction between the natural properties of lignocellulose (*e.g*., high crystallinity and hydrophilicity) and the demands of the synthesis process, which leads to technical bottlenecks such as poor structural controllability, insufficient interfacial compatibility, and difficulties in scale-up preparation in the preparation of electrolytes. The introduction of functional groups through chemical modification (*e.g*., etherification, esterification, or graft copolymerization) can reduce the polarity, while the use of silane coupling agents or interfacial compatibilizers can enhance the interfacial bonding with the substrate. However, efficient separation and purification require balancing environmental protection with cost and avoiding contamination from strong acid/base treatment. Therefore, realizing green pretreatment and preserving the nanoscale active structure of cellulose is a future concern. In addition, the research on lignocellulose-based gel electrolytes in the field of energy storage devices is still at the laboratory level, and realizing practical applications is still a challenge. Matchability of electrodes and gel electrolytes, high-rate electrochemical performance, preparation process and cost, and application of functional gel electrolytes in smart devices need to be further researched and explored.
(ii) ***Awareness of L-GPEs microscopic linkage chemistry***. The study of the linkage chemistry of lignocellulose-mediated gel electrolytes is a central breakthrough toward the pre-design of their properties and functions, which mainly includes their internal chemical cross-linking and intermolecular interactions. Notably, chemical cross-linking enables the formation of three-dimensional network structures, which improves the mechanical strength and thermal stability of the gels. However, conventional covalent cross-linking (*e.g*., epoxy cross-linking) enhances the mechanical strength but sacrifices the self-healing properties and ion mobility of the gel. In contrast, the dynamic balance of chemical bonds (*e.g*., hydrogen bonding, ionic bonding, etc.) in gels is crucial for the mechanical properties and self-healing ability of gels. Therefore, the balance between rigid covalent bonds and flexible dynamic bonds (*e.g*., hydrogen bonds, coordination bonds) deserves special attention. In addition, more attention should be paid to the side reactions at the electrode electrolyte interface, which are mainly due to the residual reactive hydroxyl groups on the cellulose surface that are easily oxidized at high voltages, which in turn triggers interfacial decomposition or lithium dendritic crystal growth, and affects the mechanical properties and interfacial compatibility of the gel electrolyte. Therefore, it is necessary to focus on dynamic interface design the future to promote the practicalization of high-safety and long-life cellulose-based energy storage devices.
(iii) ***Awareness of characterization techniques for specific applications of L-GPEs.*** The application of lignocellulose-mediated gel electrolytes in energy storage has demonstrated significant advantages, and their future development is highly dependent on the innovation and breakthrough of characterization techniques. Attention needs to be paid to the advances in *in situ* characterization techniques. Lignocellulose-mediated gel polymer electrolytes are highly dynamic in terms of structural evolution, ion migration, and interfacial reactions during the charging and discharging process, and it is difficult to capture the key transient behaviors by traditional *non-*in situ characterization means. First, the innovation of in situ characterization techniques is imminent, such as in situ EIS and cyclic voltammetry (CV), which can monitor the performance changes of the electrolyte under operating conditions in real-time and provide a direct basis for optimizing energy storage devices. Secondly, the integration of multi-scale characterization is of interest. Combining microstructural characterization (*e.g*., scanning electron microscopy (SEM) and transmission electron microscopy (TEM)) with macroscopic performance testing achieves a comprehensive understanding from micro to macro. Meanwhile, the combination of multi-scale simulation and experimental validation allows for more accurate prediction and optimization of electrolyte performance. In addition, advanced artificial intelligence and machine learning provide sufficient data on gel electrolytes with different processes and compositions. More importantly, the development of environmentally friendly characterization methods is recommended to further reduce harmful chemical reagents and energy consumption. These technological breakthroughs will drive lignocellulose-mediated gel electrolytes from the laboratory to large-scale applications, especially in the fields of high-security solid-state batteries and wearable electronics, which show great potential.
